# A multi-phase, multi-method assessment of national COVID-19 vaccination performance with equity analysis

**DOI:** 10.1038/s41598-026-39677-z

**Published:** 2026-02-10

**Authors:** Mohammadreza Rasouli, Amirreza Salehi, Majid Rafiee, Omid Fatahi Valilai

**Affiliations:** 1 Department of Industrial Engineering, Sharif University of Technology, Tehran, Iran; 2 Emerging Technologies in Industrial Engineering (EITIE) Research Group, School of Business, Social & Decision Sciences, Constructor University Bremen, Campus Ring 1, 28759 Bremen, Germany

**Keywords:** COVID-19, Multi-criteria decision-making (MCDM), Machine learning, Vaccination strategies, Decision-support system, Health care, Mathematics and computing

## Abstract

The COVID-19 pandemic, originating in Wuhan, China, rapidly escalated into a global crisis, straining public health systems and economies. This study proposes a data-driven framework to evaluate the response performance of 143 countries across three phases: pre-vaccination, vaccination, and post-vaccination. Countries are clustered using K-means clustering based on pandemic-related indicators, and indicator importance is derived using an objective weighting method that accounts for variability and interdependence. Three Multi-Criteria Decision-Making (MCDM) methods (MACONT, COCOSO, and EDAS) are then applied to rank countries within clusters. The framework integrates diverse measures, including healthcare capacity, economic resilience, and the Risk INFORM COVID-19 index, enabling a holistic evaluation. Results reveal notable differences in country performance over time, with early vaccination rollout and strict public health policies associated with more stable rankings. Key determinants include the strength of the healthcare system, economic adaptability, and equitable vaccine distribution. An equity analysis using the Gini coefficient highlights significant disparities in global vaccine access, emphasizing the urgency of coordinated policy action. Finally, by tracking country movements across clusters, the study provides insights into evolving national responses. It concludes with recommendations for improving global preparedness and building more resilient, equitable systems for future health emergencies.

**Supplementary Information:**

The online version contains supplementary material available at 10.1038/s41598-026-39677-z.

## Introduction

 In late December 2019, several cases of pneumonia of unknown etiology emerged in Wuhan, Hubei Province, China^1,2^. Subsequent epidemiological investigations identified the causative agent as a novel coronavirus (SARS-CoV-2), leading the World Health Organization (WHO) to designate the disease as COVID-19. Given its rapid human-to-human transmission and escalating global spread, the WHO declared the outbreak of a Public Health Emergency of International Concern (PHEIC) on January 30, 2020. As infections surged across multiple continents, COVID-19 was officially classified as a global pandemic on March 11, 2020^3^. The COVID-19 pandemic has had profound and multifaceted global repercussions, disrupting public health systems, economies, and social structures on an unprecedented scale^4,5^. As of recent estimates, over 704 million individuals have been officially diagnosed with COVID-19, with approximately 7 million fatalities attributed to the disease^6^. The pandemic triggered the most severe global economic recession since the Great Depression, while also disrupting education systems and significant global events worldwide^7,8^. As COVID-19 spread globally, governments implemented non-pharmaceutical interventions (NPIs) to mitigate viral transmission and safeguard public health. These measures included the closure of educational institutions and workplaces, the cancellation of large-scale public gatherings, the enforcement of mask mandates, and the implementation of stay-at-home orders. The development and large-scale deployment of COVID-19 vaccines represented a transformative milestone in the global response to the pandemic^9^. As the virus spread rapidly and NPIs were extensively implemented, mass vaccination emerged as the most effective strategy for reducing disease severity and mortality, enabled by unprecedented advances in mRNA and vector-based vaccine technologies that allowed rapid global deployment by late 2020^10^. A critical objective in pandemic management has been achieving herd immunity, which requires a substantial proportion of the population to develop immunity, either through vaccination or prior infection, to effectively disrupt transmission chains^11^. While estimates vary based on vaccine efficacy, virus transmissibility, and the evolution of new variants, initial projections suggested that 60–80% of the population needed to be immunized to establish community-wide protection^12^. However, challenges such as waning immunity, vaccine hesitancy, and the emergence of highly transmissible variants have complicated the long-term achievement of herd immunity, underscoring the need for booster vaccination programs, continuous genomic surveillance, and adaptive public health policies to sustain pandemic control efforts^13,14^.

Despite extensive global efforts to contain the spread of COVID-19 through NPIs and large-scale vaccination initiatives, the effectiveness of pandemic response strategies has varied significantly across countries. Differences in healthcare infrastructure, governmental policies, public adherence to preventive measures, and socio-economic resilience have contributed to disparities in infection rates, mortality trends, and economic recovery^15,16^. Among these factors, the efficiency of national vaccination programs has played a decisive role in shaping the pandemic’s trajectory, influencing not only the speed of viral containment but also the burden on healthcare systems and the long-term socio-economic impact^17^. Given these variations, a systematic evaluation of countries’ pandemic management performance, particularly in the implementation and distribution of vaccines, is essential for identifying best practices, highlighting policy inefficiencies, and addressing critical areas for improvement^18,19^. To address the identified gaps in the existing literature and provide a structured evaluation of national pandemic responses, this study is guided by the following research questions:

RQ1: How can countries be systematically grouped based on their structural, healthcare, economic, and vulnerability characteristics to enable fair and meaningful performance comparison during a global pandemic?

RQ2: How does national COVID-19 response performance evolve across the pre-vaccination, vaccination, and post-vaccination phases when assessed using a hybrid Machine Learning (ML) and Multi-Criteria Decision-Making (MCDM) framework?

RQ3: Which factors and criteria play the most influential role in determining country performance within each phase and cluster?

RQ4: To what extent do inequities in vaccination coverage and response capacity persist across countries, and how do these disparities affect overall pandemic performance rankings?

The contributions of this work are summarized as follows:

- Unlike prior research, which often adopts static evaluation frameworks, this study introduces a dynamic approach by assessing country performance across three distinct phases: pre-vaccination, vaccination, and post-vaccination.
- This study is the first to integrate Risk INFORM COVID-19 criteria with conventional pandemic response indicators, thereby providing a more comprehensive evaluation framework.
- This study develops a hybrid Decision Support System (DSS) that integrates ML with MCDM techniques to enhance predictive accuracy and decision-making in pandemic response. By combining MACONT, COCOSO, and EDAS, the proposed approach ensures a more robust and reliable evaluation of countries’ response effectiveness.

Based on these contributions, the primary objective of this study is to develop a robust, data-driven decision-support framework for dynamically and equitably evaluating national COVID-19 response performance. Specifically, the study aims to (i) enable fair cross-country comparison through clustering, (ii) assess performance evolution across distinct pandemic phases using multiple MCDM techniques, and (iii) identify key drivers and inequities that can inform evidence-based policy interventions and future pandemic preparedness.

The remainder of this paper is organized as follows. “Literature review” reviews the related literature on ML, MCDM, and hybrid ML–MCDM approaches applied to the evaluation of COVID-19 responses. “Methodology” presents the proposed methodology and data sources. “Results” reports the empirical results and comparative analysis. “Discussion” discusses the findings and their managerial and policy implications. Finally, “Conclusion” concludes the paper and outlines directions for future research.

## Literature review

This section reviews existing studies on COVID-19 response evaluation, focusing on ML approaches, MCDM methods, and integrated ML–MCDM frameworks applied to pandemic management and vaccine-related decision-making.

### MCDM-Based pandemic response studies

In this context, MCDM methodologies provide a structured framework for evaluating and comparing national responses to public health crises. These approaches incorporate key performance indicators such as healthcare system capacity, policy effectiveness, emergency responsiveness, and vaccination coverage, enabling a systematic assessment of how different countries have managed the COVID-19 pandemic. Given that a combination of interdependent factors influences effective pandemic management, applying these methodologies enables the identification of successful strategies, the recognition of policy shortcomings, and the development of evidence-based recommendations to enhance global preparation for future health emergencies. A comprehensive analysis using these decision-making frameworks enables policymakers to extract key lessons from high-performing countries, refine ineffective policies, and develop more adaptive and data-driven strategies for future pandemic management^18^. O.S. Albahri et al. examined COVID-19 vaccine distribution as an MCDM problem, focusing on distribution criteria, criteria importance, and data variation. They utilized the Pythagorean fuzzy decision by opinion score method (PFDOSM) for ranking vaccine recipients but noted its limitation in implicit criteria weighting. To address this, they proposed the fuzzy-weighted zero-inconsistency (FWZIC) method, which assigns explicit weights while ensuring zero inconsistency, though it may still face challenges in handling vagueness^20^. Sarwar, Adnan, et al. investigated the willingness of the general public in Pakistan to receive the COVID-19 vaccine using the Analytic Hierarchy Process (AHP). Their study identified key determinants influencing vaccination decisions, including cues to action, perceived benefits, government recommendations, and perceived stress, which positively impacted willingness. Conversely, individual decision, vaccine origin, resistance to change, and perceived barriers were significant obstacles to vaccine uptake. The authors emphasized the need for government-led communication campaigns to reinforce the benefits of vaccination and improve public acceptance^21^. Alemdar, Kadir Diler, et al. addressed the challenge of selecting optimal vaccination centers in metropolitan areas, where high population density and case numbers complicate site allocation. They proposed a three-step approach combining expert-defined criteria, the Analytic Hierarchy Process (AHP) for weighting, and the Geographic Information System (GIS) for spatial analysis. Applying this method to Bağcılar, Istanbul, they identified three suitable areas for vaccination centers. Their approach offers a scientific and strategic framework for optimizing vaccine distribution and improving pandemic response^22^. Similarly, Seker et al.^23^ introduced an IVq-ROF-COPRAS model for COVID-19 risk assessment, aiding governments in prioritizing response measures through sensitivity and comparative analysis. MCDM techniques have also been employed in financial and industrial performance evaluations during the pandemic. Makki and Alqahtani^24^ assessed financial resilience in Saudi Arabia’s energy sector, while Ergülen and Çalık^25^ analyzed Turkey’s top 500 industrial enterprises, identifying key factors contributing to economic stability amid the crisis.

### ML applications in COVID-19 analysis

In addition to MCDM methodologies, data-driven approaches, and ML techniques have become essential tools for conducting a more comprehensive and precise assessment of national responses to public health crises^26^. The management of the COVID-19 pandemic is inherently complex, as it involves interdependent healthcare, economic, and social factors that cannot be fully captured through traditional analytical frameworks. Data-driven models enable the systematic analysis of large-scale data on policy interventions, healthcare system performance, vaccination effectiveness, and socio-economic trends, thereby identifying hidden patterns and relationships that may not be evident through conventional evaluation methods. Moreover, these approaches enhance the accuracy of assessments, minimize subjective biases, and facilitate a more objective comparison of pandemic management strategies across countries^27^. Ibrahim et al.^28^ demonstrated that ensemble learning models significantly enhance daily COVID-19 case forecasting, while Khattab et al.^29^ examined the impact of Focal Loss in deep learning-based medical imaging, addressing class imbalance in pneumonia and COVID-19 detection. These advancements highlight how ML algorithms improve epidemiological forecasting and medical diagnosis, directly influencing vaccination and treatment strategies. In contrast, MCDM techniques have been widely applied to assess pandemic control policies, rank national responses, and support strategic decision-making^30^. Moosazadeh et al.^31^ validated a hybrid ML model to evaluate COVID-19 vulnerability across U.S. counties, providing insights into the effectiveness of public health interventions. Zgheib et al.^32^ explored AI and IoT-driven solutions to improve real-time decision-making and crisis response. Cheong, Queena, et al. examined regional disparities in COVID-19 incidence across U.S. counties, highlighting the influence of sociodemographic factors on the unequal spread of the disease. They emphasized the need for accurate predictive modeling to help public health officials mitigate healthcare burdens.

### Hybrid ML–MCDM frameworks

Integrating ML techniques with MCDM-based evaluation frameworks enables a more robust, predictive, and adaptive approach to analyzing national responses. This combination not only facilitates structured cross-country comparisons but also helps identify key determinants of effective pandemic control measures. By leveraging these advanced methodologies, policymakers can gain data-driven insights to refine public health strategies, optimize resource allocation, and strengthen preparedness for future global health emergencies^33^.

Since the outbreak of COVID-19, extensive research has been conducted across various disciplines, employing diverse analytical methodologies. These studies can be broadly categorized into three main approaches: ML-based methods, MCDM-based methods, and integrated ML-MCDM approaches, each offering distinct perspectives on analyzing and managing the pandemic ML-based approaches have been widely utilized to model disease transmission patterns, predict infection rates, optimize resource allocation, and assess vaccine effectiveness^34^. Hadi et al.^35^ introduced CORONA-NET, a lightweight deep learning model that integrates CNN, DWT, and LSTM to analyze chest X-ray images. Similarly, Chakraborty and Mali^36^ proposed SUFEMO, a segmentation technique for radiological images that combines electromagnetism-like optimization, type-2 fuzzy logic, and superpixels, improving accuracy while reducing computational costs. Beyond medical applications, ML-based models have been employed to assess pandemic response strategies. Additionally, despite widespread vaccine availability, vaccination rates have stagnated, reinforcing the importance of predictive models in identifying key factors influencing vaccine uptake^37^. Ong, Edison, et al. explored COVID-19 vaccine development using Vaxign and Vaxign-ML, ML-based reverse vaccinology tools. Their analysis identified six potential vaccine candidates, including the S protein and five non-structural proteins (nsp3, 3CL-pro, and nsp8-10), which play crucial roles in viral adhesion and host invasion. Among them, nsp3, which has not been widely studied in coronavirus vaccines, showed high protective antigenicity and strong conservation across SARS-CoV-2, SARS-CoV, and MERS-CoV. The authors proposed an **‘**Sp/Nsp cocktail vaccine**’** combining structural and non-structural proteins to enhance immune response effectiveness^38^. Predictive modeling has also played a crucial role in pandemic management. Alkan and Kahraman^39^ employed the intuitionistic fuzzy TOPSIS method to compare national strategies under uncertainty, demonstrating how fuzzy-based decision models enhance crisis response. Similarly, Ecer and Pamucar^40^ applied the MARCOS technique within an intuitionistic fuzzy environment to prioritize insurance companies’ contributions to healthcare during the pandemic. A significant challenge in pandemic response has been vaccine distribution and prioritization, an area where MCDM models have played a critical role. Hezam et al.^41^ introduced an AHP-TOPSIS model to allocate COVID-19 vaccines, considering factors such as age, health status, gender, and occupation type. Their findings emphasize the need for structured decision-making models to ensure equitable and efficient vaccine allocation. MCDM has also been utilized to assess the resilience of pharmaceutical supply chains. Shanker et al.^42^ applied a hybrid DEMATEL-ANP framework to analyze supply chain disruptions, offering strategies for resilience against future crises. Salehi et al.^43^ used entropy-based MCDM to assess healthcare workers’ stress levels, while Ahmad et al.^44^ developed a fuzzy MCDM framework to evaluate mental health stressors during lockdowns. Additionally, MCDM approaches have been employed to rank countries based on their pandemic response performance. Özgür and Güre^45^ ranked 18 OECD countries using multi-criteria decision models, while Matsumoto et al.^46^ applied DEA and the Malmquist-Luenberger index to assess the economic and environmental consequences of COVID-19. The use of hybrid MCDM models has further expanded the applicability of these methodologies in pandemic-related decision-making. Mete et al.^47^ developed a two-phase MCDM framework, integrating the Risk INFORM COVID-19 Index with COPRAS and MC-BWM methods, to rank 29 countries based on 40 pandemic-related indicators.

More recently, ML-MCDM hybrid models have emerged as powerful tools for combining predictive analytics with structured evaluation techniques, offering a more adaptive, context-aware decision-making framework. One of the key applications of integrated ML-MCDM models is in ranking and benchmarking pandemic response strategies. Mohammed et al.^48^ developed an ML-MCDM framework for evaluating diagnostic models, integrating TOPSIS for ranking and Entropy for weight assignment. Similarly, Aggarwal et al.^49^ proposed an SEIR-based multi-criteria decision support system, leveraging ML predictions alongside MCDM’s structured evaluation. In diagnostics, Chowdhury et al.^50^ combined ML and MCDM approaches to classify COVID-19 cases using cough sound analysis, demonstrating how ML’s pattern recognition can be systematically validated using MCDM frameworks. Aydın and Yurdakul^51^ introduced a three-stage ML-MCDM model to assess 143 countries’ pandemic response efficiency, using DEA for efficiency analysis, ML-based clustering, and MCDM for ranking influential factors. Table 1 summarizes the key studies on COVID-19 response evaluation, highlighting their methodologies, contributions, findings, and implications, with our research positioned in the final row to emphasize its unique approach.

**Table 1:** Summary of literature on COVID-19 response evaluation.

Author(s)	Year	Contribution	Findings	Implications
O.S. Albahri et al.^20^	2021	Examined vaccine distribution as an MCDM problem using PFDOSM and proposed FWZIC for explicit weighting	PFDOSM ranks vaccine recipients; FWZIC ensures zero inconsistency in weighting	Enhances equitable vaccine distribution through structured decision-making frameworks
Sarwar, Adnan, et al.^21^	2021	Investigated public willingness for COVID-19 vaccination in Pakistan using AHP	Identified cues to action, perceived benefits, and government recommendations as key drivers; individual decisions and vaccine origin as barriers	Supports government-led campaigns to improve vaccine acceptance and uptake
Alemdar, Kadir Diler, et al.^22^	2021	Proposed a three-step MCDM approach combining AHP and GIS for vaccination center selection in metropolitan areas	Identified three suitable areas for vaccination centers in Bağcılar, Istanbul	Provides a strategic framework for optimizing vaccine distribution in high-density areas
Hadi et al.^35^	2020	Introduced CORONA-NET, integrating CNN, DWT, and LSTM for chest X-ray analysis	High diagnostic accuracy in detecting COVID-19 from chest X-rays	Improves medical imaging for rapid and accurate COVID-19 diagnosis
Chakraborty and Mali^36^	2020	Proposed SUFEMO, combining electromagnetism-like optimization, type-2 fuzzy logic, and superpixels for radiological imaging	Improved accuracy in image segmentation with reduced computational costs	Enhance diagnostic imaging efficiency for COVID-19 and related diseases
Moosazadeh et al.^31^	2021	Validated a hybrid ML model to assess COVID-19 vulnerability across U.S. counties	Sociodemographic factors influence vulnerability to COVID-19	Informs targeted public health interventions in vulnerable U.S. regions
Zgheib et al.^32^	2021	Explored AI and IoT-driven solutions for real-time crisis response	AI and IoT improve decision-making and crisis response capabilities	Supports real-time policy adjustments and resource allocation during pandemics
Cheong, Queena, et al.^37^	2021	Examined regional disparities in COVID-19 incidence across U.S. counties	Sociodemographic factors drive unequal disease spread	Guides public health officials in addressing healthcare disparities through predictive modeling
Ong, Edison, et al.^38^	2021	Used Vaxign and Vaxign-ML to identify vaccine candidates, proposing a Sp/Nsp cocktail vaccine	Identified six vaccine candidates, including S protein and nsp3, with high antigenicity	Advances in vaccine development to enhance immune responses against coronaviruses
Ibrahim et al.^28^	2021	Demonstrated ensemble learning for daily COVID-19 case forecasting	Ensemble models significantly improve forecasting accuracy	Enhances epidemiological forecasting for better pandemic planning and response
Khattab et al.^29^	2021	Examined Focal Loss in deep learning for medical imaging to address class imbalance	Improved detection of pneumonia and COVID-19 in imbalanced datasets	Strengthens diagnostic accuracy in medical imaging for pandemic-related diseases
Alkan and Kahraman^39^	2021	Employed intuitionistic fuzzy TOPSIS to compare national strategies under uncertainty	Fuzzy-based models enhance crisis response evaluation	Supports robust decision-making for national pandemic strategies
Ecer and Pamucar^40^	2021	Applied MARCOS in an intuitionistic fuzzy environment to prioritize insurance contributions to healthcare	Ranked insurance companies’ contributions effectively	Guides healthcare resource allocation during pandemics
Hezam et al.^41^	2021	Developed an AHP-TOPSIS model for equitable COVID-19 vaccine allocation	Equitable allocation based on age, health status, and occupation	Promotes fair and efficient vaccine distribution strategies
Shanker et al.^42^	2021	Applied hybrid DEMATEL-ANP to analyze pharmaceutical supply chain disruptions	Identified strategies for supply chain resilience	Enhances supply chain preparedness for future health crises
Salehi et al.^43^	2021	Used entropy-based MCDM to assess healthcare workers’ stress levels	Quantified stress factors impacting healthcare workers	Informs policies to support healthcare workers’ well-being during pandemics
Ahmad et al.^44^	2021	Developed a fuzzy MCDM framework to evaluate mental health stressors during lockdowns	Identified key mental health stressors	Guides mental health support strategies during public health crises
Özgür and Güre^52^	2021	Ranked 18 OECD countries using multi-criteria decision models	Provided comparative rankings of national responses	Offers insights for benchmarking pandemic strategies across OECD countries
Matsumoto et al.^46^	2021	Applied the DEA and Malmquist-Luenberger index to assess economic and environmental impacts	Evaluated economic and environmental consequences of COVID-19	Informs policies balancing economic and ecological outcomes during pandemics
Mete et al.^47^	2022	Integrated Risk INFORM COVID-19 Index with COPRAS and MC-BWM to rank 29 countries	Ranked countries based on 40 pandemic-related indicators	Provides a structured framework for evaluating global pandemic response
Seker et al.^23^	2022	Introduced the IVq-ROF-COPRAS model for COVID-19 risk assessment	Prioritized response measures through sensitivity analysis	Aids governments in prioritizing effective pandemic response strategies
Makki and Alqahtani^24^	2022	Assessed financial resilience in Saudi Arabia’s energy sector using MCDM	Identified factors contributing to financial stability	Supports economic resilience strategies in critical sectors during crises
Ergülen and Çalık^25^	2022	Analyzed Turkey’s top 500 industrial enterprises using MCDM	Identified key factors for economic stability	Guides industrial policy for economic resilience during pandemics
Mohammed et al.^48^	2022	Developed an ML-MCDM framework using TOPSIS and Entropy for diagnostic model evaluation	Ranked diagnostic models effectively	Enhances diagnostic model validation for pandemic management
Aggarwal et al.^49^	2022	Proposed an SEIR-based multi-criteria decision support system with ML and MCDM	Integrated predictive and evaluative approaches for response assessment	Supports comprehensive decision-making for pandemic control
Chowdhury et al.^50^	2022	Combined ML and MCDM for COVID-19 case classification using cough sound analysis	Improved case classification through pattern recognition and validation	Advances in non-invasive diagnostic methods for COVID-19
Aydin and Yurdakul^51^	2022	Introduced a three-stage ML-MCDM model using DEA, ML clustering, and MCDM for 143 countries	Assessed efficiency and ranked national responses	Provides a robust framework for evaluating and ranking global responses
This study	2025	Developed a hybrid ML-MCDM framework integrating K-means, MACONT, COCOSO, EDAS, and Risk INFORM COVID-19 for 143 countries across three phases	Dynamic evaluation across pre-vaccination, vaccination, and post-vaccination phases, with vaccine equity analysis and policy insights	Offers a comprehensive, dynamic framework for evaluating global pandemic responses, enhancing policy recommendations for future health emergencies

One widely recognized framework for assessing pandemic risk is the Risk INFORM COVID-19 Index, developed by the European Commission. This index integrates 40 indicators across three key dimensions: vulnerability, inability to cope, and hazards/exposure, providing a structured approach to evaluating country-specific risks. However, traditional risk indices like INFORM rely primarily on static, arithmetic-based methods, which may not fully capture the pandemic’s dynamic, evolving nature. Furthermore, existing studies often focus on a limited set of criteria and do not incorporate ML techniques for deeper insights^47^. By addressing these key research gaps, this study contributes to the existing literature on pandemic management and provides actionable insights for policymakers and global health organizations.

## Methodology

The COVID-19 pandemic has significantly impacted the world, necessitating swift and effective responses from nations. Evaluating countries’ pandemic performance is crucial to understanding their success in controlling the virus’s spread, implementing preventive measures, and protecting public health. To analyze and evaluate countries’ performance in managing the COVID-19 crisis, a structured, multidimensional approach is essential. This section presents the methodologies and techniques employed in this study for data processing, criteria analysis, and country ranking. These methods have been carefully selected to address the problem and ensure accurate, reliable results comprehensively.

### Proposed framework

In this study, we propose a hybrid DSS designed to address the multi-dimensional complexity of pandemic assessment. The framework integrates unsupervised ML with MCDM to ensure a robust and unbiased evaluation. First, K-means clustering is employed to handle the structural heterogeneity of nations, ensuring that countries are evaluated against peers with similar socio-economic and healthcare profiles rather than through a ‘one-size-fits-all’ global ranking. Following stratification, the CRITIC method is utilized to derive objective weights for the evaluation criteria. Unlike subjective weighting methods that rely on expert opinion, CRITIC leverages the intrinsic contrast and correlation within the data to capture the accurate statistical information content of each indicator. Finally, to mitigate the methodological bias inherent in any single ranking technique, a consensus-based ranking is generated using an ensemble of three distinct MCDM methods: MACONT, COCOSO, and EDAS. This triangulation approach, combining comprehensive normalization (MACONT), compromise solution theory (COCOSO), and deviation-based evaluation (EDAS), ensures that the final rankings are mathematically stable and reflect a balanced view of national performance.

**Fig. 1:**
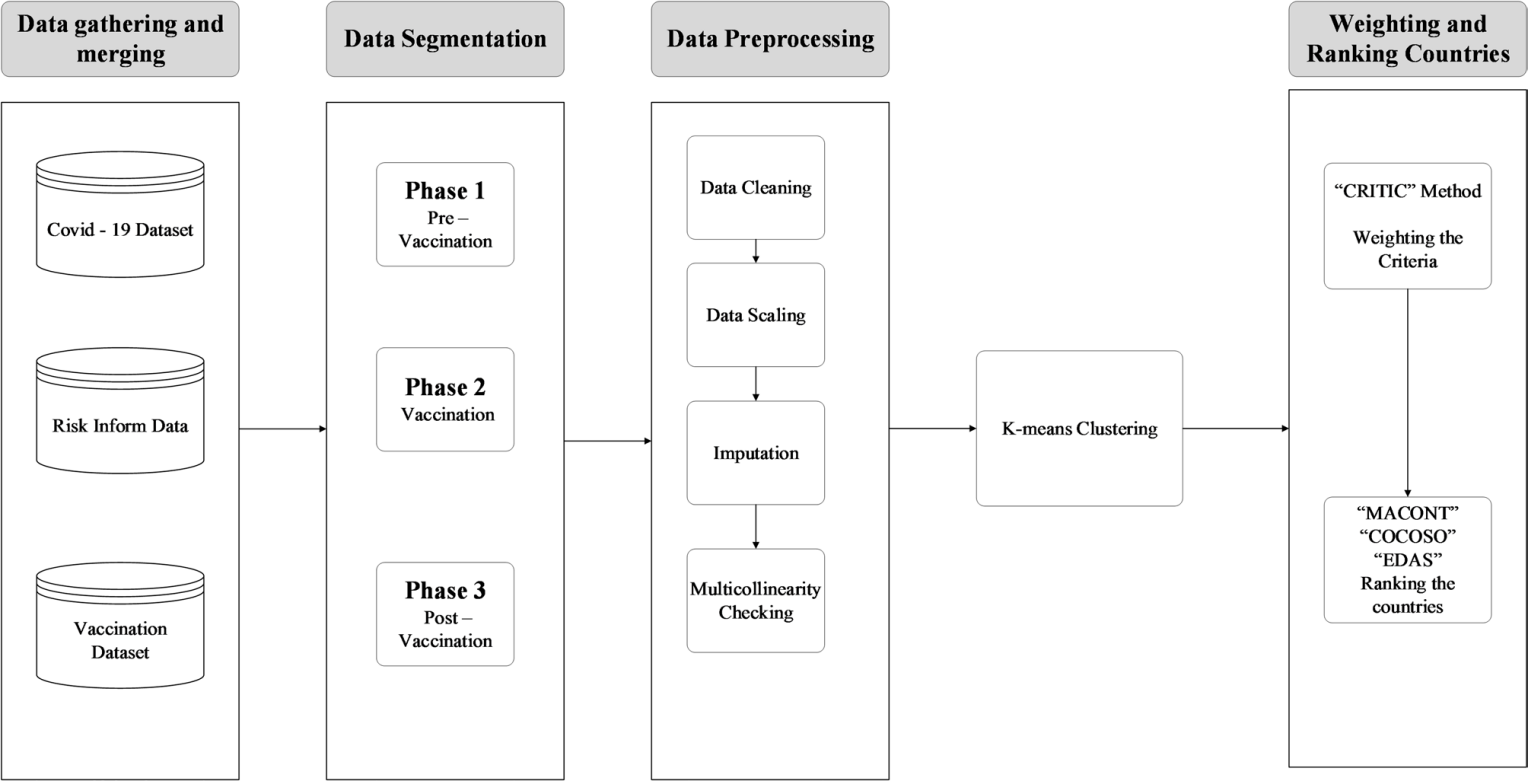
Proposed framework illustration.

As illustrated in Fig. 1, the proposed framework follows a structured sequence of steps. In Step 1, data are collected from multiple reputable databases and integrated into a unified dataset. In Step 2, the dataset is segmented into three distinct phases (pre-vaccination, vaccination, and post-vaccination) to account for temporal differences in pandemic responses. In Step 3, data preprocessing is performed, including cleaning, transformation, normalization, and handling missing values using the MICE method, ensuring data consistency and reliability.

In Step 4, the K-means clustering algorithm partitions countries into distinct groups. Given the multi-dimensional nature of the dataset encompassing health, economic, and social metrics, K-means was selected for its computational efficiency and its statistical capability to minimize within-cluster variance. Although standard K-means is sensitive to outliers, this study mitigates that risk through the rigorous preprocessing procedures established in Step 3 (detailed in “ML applications in COVID-19 analysis”). Specifically, the application of a Z-score threshold (± 3) to remove technical artifacts and logarithmic transformations to normalize skewed distributions ensures that the dataset is statistically suitable for centroid-based clustering. Consequently, the resulting clusters reflect robust structural similarities in national pandemic baselines rather than inconsistencies caused by extreme anomalies. Following clustering, Step 5 employs a hybrid MCDM framework designed to address specific data characteristics, particularly the presence of conflicting criteria and statistical interdependencies. The CRITIC method is used for weighting because, unlike the Entropy method, which only considers data dispersion, CRITIC accounts for both contrast intensity (standard deviation) and conflict (correlation) between criteria. This is statistically vital for this study, as pandemic indicators (like GDP Per Capita and Healthcare Capacity) often exhibit significant multicollinearity; ignoring these correlations would lead to biased weight distribution.

Subsequently, a composite ranking is derived using three distinct MCDM techniques, MACONT, COCOSO, and EDAS, selected to mitigate methodological bias and handle data heterogeneity. MACONT is employed for its comprehensive normalization technique, which effectively standardizes the varying scales of epidemiological and economic data. COCOSO is integrated for its stability, combining additive and multiplicative aggregation strategies to reduce the impact of extreme values. Finally, EDAS is included for its distance-based approach, which evaluates performance based on positive and negative deviations from the average solution rather than an absolute ideal, making it robust against the outliers inherent in pandemic data [68]. This triangulation of methods ensures a holistic evaluation that is not sensitive to the algorithmic limitations of a single technique. Finally, in Step 6, the final ranking of countries is determined by aggregating the rankings from the three MCDM methods. An averaging approach is employed to reduce biases introduced by any single method, providing a more balanced evaluation of national performance during the pandemic.

This structured and data-driven approach not only enhances the interpretability of the evaluation process but also provides a reliable methodology for assessing and comparing national responses to the COVID-19 crisis across different phases of the pandemic.

### Data definition and preprocessing

This study investigates and evaluates the performance of 231 countries during the COVID-19 pandemic using data from three datasets. These datasets were sourced from reputable organizations, including the WHO, the World Bank, Our World in Data, the United Nations, and Kaggle. The collected data includes indicators related to the pandemic and vaccination efforts, enabling a comprehensive analysis of the situation across countries. The first dataset consists of time-series data, including daily records of COVID-19-related indicators such as new tests, new cases, new deaths, and other pandemic-related metrics. The second dataset comprises both static and time-series data on vaccination efforts, including total vaccinations, the first vaccination date, and other relevant indicators. The third dataset contains data from the Risk INFORM COVID-19 Index, which assesses a country’s vulnerability to the pandemic, its response capacity, and exposure risk factors. Although the initial data collection covered 231 countries, many had incomplete or missing information across key indicators. To ensure robustness and comparability, only 143 countries with sufficient, reliable data were retained for further analysis. By integrating these three datasets, the study ensures a holistic approach to assessing the pandemic’s impact and response strategies implemented worldwide. The timeframe under investigation spans from May 1, 2020, to August 14, 2024, and is divided into three distinct phases that reflect the progression of the pandemic and vaccination efforts:

- Pre-vaccination phase (May 1, 2020–December 8, 2020): The period before the initiation of vaccination campaigns.
- Vaccination phase (December 8, 2020–August 4, 2022): The period during which vaccination programs were actively implemented.
- Post-vaccination phase (August 4, 2022–August 14, 2024): The period after widespread vaccination, assessing long-term pandemic management.

Although countries did not enter the vaccination era simultaneously, each phase in this study is defined using globally recognized epidemiological milestones rather than calendar dates, ensuring conceptual comparability despite temporal asynchrony. Specifically, adopting country-specific timelines would result in evaluating nations under disparate global epidemiological conditions like comparing one country’s performance during the milder initial strains against another’s during the Delta variant surge. Such temporal misalignment would invalidate cross-country comparisons by introducing confounding variables related to viral transmissibility and pathogenicity. By anchoring phases to global milestones, we hold the external environment constant standardizing the viral threat level, global economic context, and available scientific knowledge across all nations. This approach isolates national response performance from the confounding effects of evolving viral mutations, ensuring that the comparison remains methodologically robust even though it may expose structural inequities in resource access (as will be discussed in “Conclusion”). As mentioned in the introduction, achieving herd immunity against viruses requires 60–80% of a country’s population to be vaccinated. Therefore, we analyzed vaccination data to determine the time each country took to reach this threshold. However, some countries exhibited abysmal vaccination rates, requiring significantly longer times to achieve herd immunity. Consequently, we defined the end of the vaccination phase as the point when 90% of countries had reached the herd immunity threshold. Based on this criterion, the vaccination phase was concluded on August 4, 2022. Additionally, as previously noted, the United Kingdom was the first country to initiate a nationwide COVID-19 vaccination program. Therefore, we set the start of the vaccination phase as the date of the first nationwide vaccination in the UK, ensuring an accurate representation of the global vaccination timeline. This division allows for a structured examination of how different countries responded to and managed the pandemic over time. All datasets are available in Appendix A. Following data collection, a rigorous diagnostic procedure was employed to assess the extent and randomness of missing data across all countries and variables. To ensure the robustness of the imputation process and mitigate the risks associated with high missingness, a strict exclusion threshold was applied: countries or variables exhibiting more than 30% missing values were removed from the dataset. This threshold ensures that the remaining data possesses sufficient observed information to support valid statistical inferences. Before imputation, the missingness mechanism was evaluated to determine the appropriateness of multivariate techniques. Little’s Missing Completely at Random (MCAR) test was conducted, and inspection of missing data patterns suggested a Missing at Random (MAR) mechanism, thereby justifying the use of Multiple Imputation by Chained Equations (MICE)^52^. MICE was implemented using the Predictive Mean Matching (PMM) method, which preserves the distributional characteristics of the data and ensures that imputed values remain within plausible ranges. The imputation model was specified with 20 iterations across five imputed datasets to ensure statistical efficiency. Convergence of the MICE algorithm was assessed by visually inspecting trace plots of the means and variances for imputed variables, confirming that the chains had stabilized and were stationary, with no discernible trends. To assess the sensitivity of the downstream clustering and ranking outcomes to the imputation assumptions, a comparative robustness check was performed using K-Nearest Neighbors (KNN) imputation^52^, Unlike MICE, which relies on the Missing at Random (MAR) assumption, KNN is a non-parametric distance-based method. The analysis demonstrated high stability in the outcomes, with a Spearman rank correlation exceeding 0.95 for the rankings and a 92% overlap in cluster assignments between the two methods. This confirms that the derived results are robust and not inextricably bound to the specific statistical assumptions of the MICE algorithm. All preprocessing and imputation procedures were executed using the Scikit-learn and Statsmodels libraries in Python.

Following imputation, a rigorous data quality assessment was conducted to identify and resolve inconsistencies and outliers. First, structural consistency was verified by cross-referencing country identifiers and demographic metrics across the three source datasets to ensure alignment. Second, a univariate outlier detection procedure was implemented using a Z-score threshold of ± 3. A critical distinction between data entry errors and genuine epidemiological extremes was operationalized through a standardized two-stage validation protocol applied consistently across all indicators. First, logical domain constraints were enforced to automatically reject technical artifacts, such as probabilities exceeding 100% or negative cumulative counts. Second, statistical outliers identified via the Z-score threshold were cross-referenced against temporal epidemiological patterns; outliers corresponding to sustained, multi-day shifts (indicative of variant waves) were retained to preserve ecological validity, while isolated, discontinuous spikes lacking temporal corroboration were treated as measurement errors and excluded. All indicators used in this study are expressed as proportionate or rate-based measures, which allow meaningful comparisons across countries regardless of population size and avoid scale-driven distortions. Finally, to ensure comparability across heterogeneous scales and mitigate the influence of remaining skewness, logarithmic transformations were applied where necessary, followed by Z-score standardization to normalize the feature space for further analysis.

All data processing and analysis were performed in Python 3.12.4. Data handling and preprocessing were implemented using Pandas and NumPy, while Scikit-learn was used for imputation, normalization, and model-based procedures. Visualizations were produced using Matplotlib and Seaborn. To ensure reproducibility, all stochastic components (including K-means initialization, MICE imputation, and data splits) were executed with fixed random seeds, and the full source code has been publicly released for independent verification. The analysis was conducted on a system equipped with an Intel Core i5-2450 M 2.5 GHz processor running Windows 10. Through this rigorous data collection and preprocessing methodology, the study ensures the reliability and robustness of the datasets, laying a strong foundation for further analysis and evaluation of pandemic management strategies across different countries.

### Feature engineering

After completing data preprocessing and cleaning, we conducted a systematic analysis of multicollinearity among the features to mitigate potential instability in the model and enhance feature selection. Multicollinearity occurs when one or more independent variables exhibit high correlation, complicating the estimation of their individual effects on the dependent variable. This issue can lead to inflated variance in regression coefficients, resulting in unstable estimates and reduced model interpretability^53^. To diagnose multicollinearity, we employed the Variance Inflation Factor ($\:VIF$), a statistical metric that quantifies the extent to which the variance of a regression coefficient is increased due to collinearity with other predictors. The $\:VIF$ For the i-th feature is computed as Eq $\:\left(1\right)$:

1 $$\:{VIF}_{i}=\:\frac{1}{1-{R}_{i}^{2}}$$

where $\:{R}_{i}^{2}\:$represents the coefficient of determination obtained from regressing the i-th feature on all other features in the dataset. Higher $\:VIF$ values indicate stronger collinearity, with commonly accepted thresholds of 5 or 10 used to identify significant multicollinearity^53,54^. In this study, features with $\:VIF$ values exceeding the selected threshold were identified and removed to improve model stability, minimize redundancy, and enhance the reliability of coefficient estimation. This preprocessing step ensures that the model remains robust and interpretable, thereby strengthening the validity of the analysis. After data cleaning and feature selection, information on 143 countries, including 48 features for the pre-vaccination phase, 55 for the vaccination phase, and 51 for the post-vaccination phase, has been used. The difference in the number of features across phases is due to the varying nature of each phase. For example, it is expected that no vaccination-related features would be present in the pre-vaccination phase. To ensure a robust evaluation that accounts for critical epidemiological and structural disparities across nations, the feature set was categorized into four distinct dimensions:

- General Health, Testing, and Immunity Context: This category captures the direct impact of the pandemic and the capacity of the healthcare system. It includes standard metrics, such as Total Cases (a proxy for natural immunity prevalence) and Total Deaths, alongside critical indicators of surveillance capacity, such as Total Tests and Positive Rate. These variables address disparities in testing availability and infection detection^13^.
- Economic Resilience: This category evaluates the financial capacity of nations to weather the crisis, incorporating variables such as GDP Per Capita, Extreme Poverty, and economic support indices.
- Social and Vulnerability Indicators: These variables assess socioeconomic conditions that influence disease spread and outcome severity, including the Human Development Index (HDI), Access to Water for Handwashing, and specific vulnerability indices derived from the Risk INFORM dataset.
- Demographic and Structural Determinants: To control intrinsic transmission dynamics and fatality risks, this category explicitly includes Population Density and Urbanization Rate (addressing transmission potential) as well as Age Structure indicators like share of population aged 65 + with underlying conditions, and children under 5.

Based on the literature review of country performance evaluation during the COVID-19 pandemic and the authors’ knowledge, this is the first study to use these variables in conjunction with other categories of variables mentioned in the literature to assess country performance. The integration of Risk INFORM COVID-19 indicators with existing datasets enabled a more comprehensive assessment of national responses. These indicators, which encapsulate vulnerability, exposure, and coping capacity, provided an additional layer of insight that had been previously overlooked in pandemic evaluations. The analysis revealed that these criteria consistently received higher weightings across all clusters and phases, underscoring their significance in assessing country performance. This integration enhances the granularity of evaluation and provides policymakers with a more data-driven framework for assessing pandemic preparedness and response strategies.

## Results

**Fig. 2:**
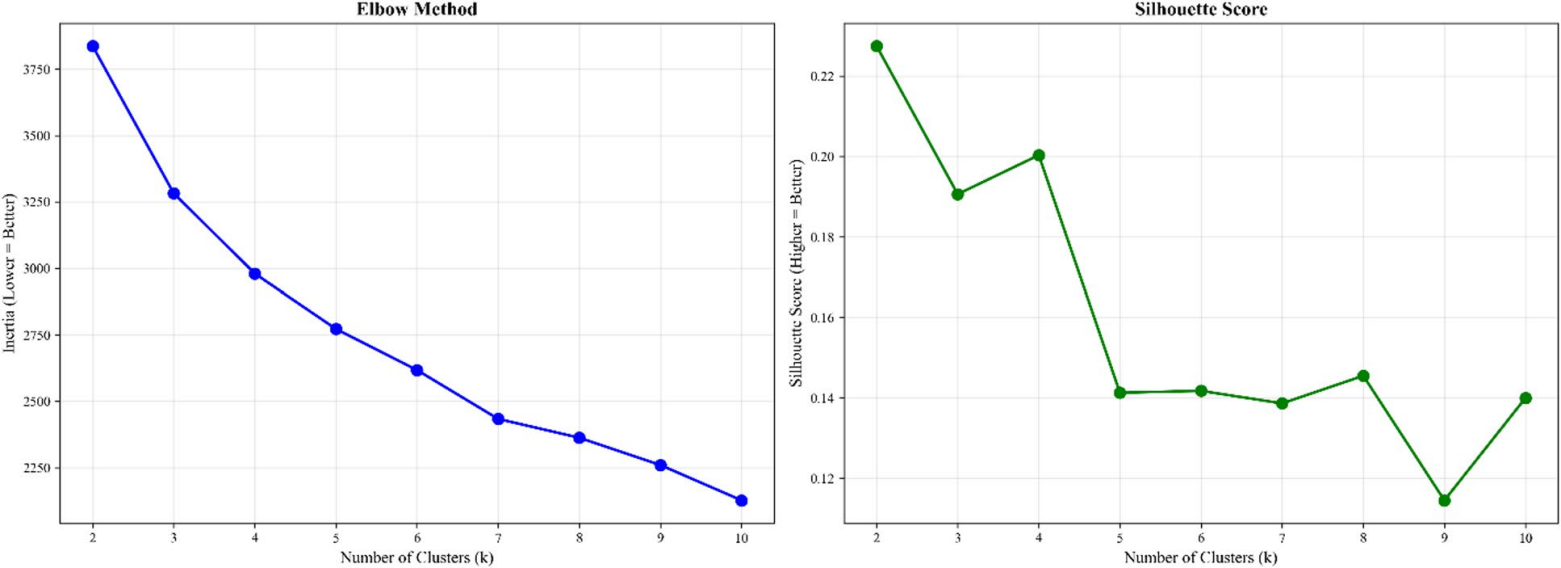
Cluster validity indices for determining best $\:k$.

After preprocessing the dataset and preparing it for clustering, highly correlated variables were identified and removed to enhance robustness and minimize redundancy. The K-means clustering algorithm was selected for its efficiency in grouping countries with similar characteristics based on health, economic, social, and demographic indicators. To identify the optimal number of clusters (k), we performed a rigorous validation analysis using the Elbow Method (Inertia) and the Silhouette Coefficient, as shown in Fig. 2. The quantitative results presented a trade-off: the Silhouette Coefficient peaked at $\:k\:=2\:\left(0.228\right)$, suggesting a broad binary classification, while the Elbow Method indicated an inflection point at $\:k\:=4$. However, selecting $\:k\:=2\:$ would oversimplify the global pandemic landscape into merely ‘high’ vs. ‘low’ performers, obscuring the critical ‘transitioning’ group of nations that exhibit moderate resilience but high vulnerability a key focus for policy intervention. Conversely,$\:k\:=4$ yielded only a marginal improvement in Silhouette score $\:\left(0.200\right)$ compared to$\:k\:=3$ $\:\left(0.191\right)$ but resulted in over-segmentation that diluted the distinctiveness of the clusters. Therefore, $\:k\:=3\:$was selected as the best equilibrium between statistical validity and practical interpretability. This tripartite structure aligns with established risk stratification frameworks (High, Medium, Low vulnerability) utilized in the Risk INFORM index, allowing for distinct, actionable policy insights without the loss of granularity associated with binary clustering or the redundancy of over-clustering.

**Fig. 3:**
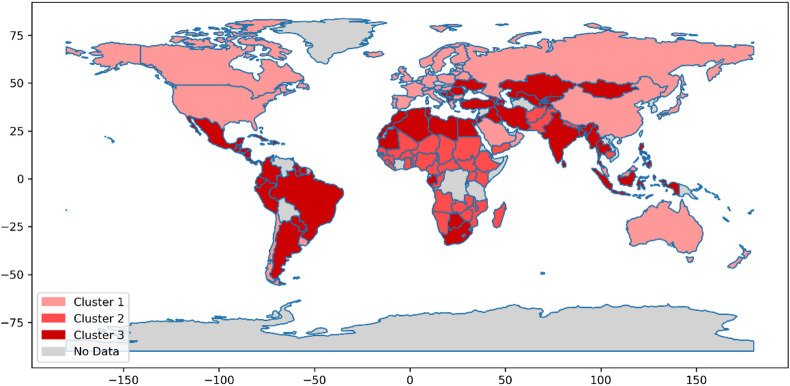
Phase 1 (pre vaccination).

**Fig. 4:**
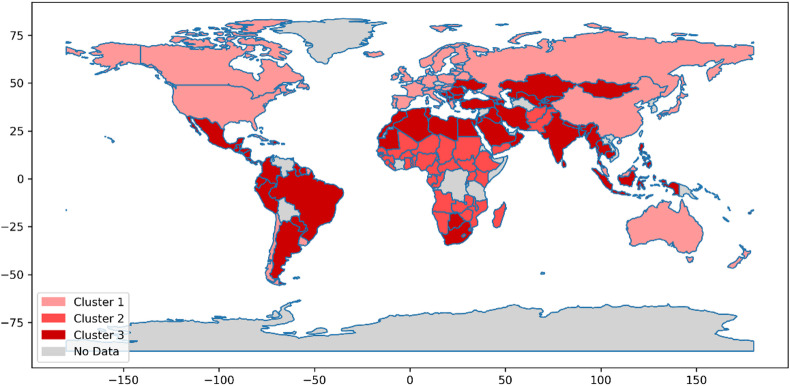
Phase 2 (vaccination).

**Fig. 5:**
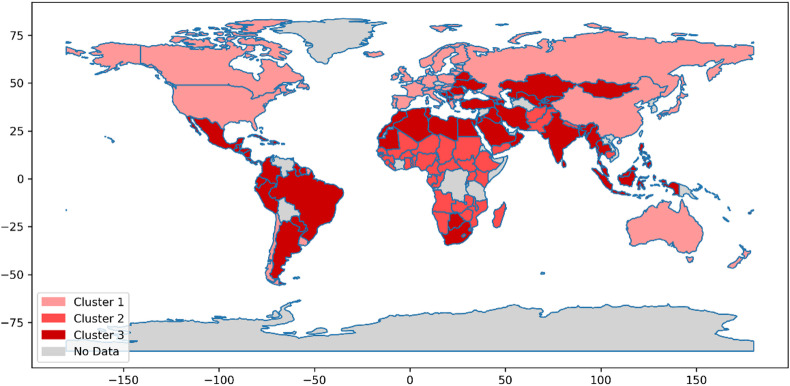
Phase 3 (post vaccination).

Figures 3, 4 and 5 comprehensively depict the trajectory of the analyzed countries across the pre-vaccination, vaccination, and post-vaccination phases. These visualizations indicate that in each time period, countries are classified into three distinct clusters. However, as the pandemic evolved, a dynamic pattern of inter-cluster movement emerged, demonstrating that countries transitioned between clusters in response to changing conditions, policies, and pandemic developments. Following the clustering process, the next step was to evaluate the performance of countries within each cluster. This evaluation was conducted using the MACONT MCDM method, ensuring a systematic and rigorous ranking process. To establish the relative importance of each criterion, the CRITIC method was applied for each phase and cluster. The number of criteria varied across the three time periods: 48 in the pre-vaccination phase, 55 during the vaccination phase, and 51 in the post-vaccination phase. The adoption of a three-cluster structure facilitated a structured comparison of national performance while accounting for variations in key pandemic-related factors. Given the dynamic nature of the crisis, this ranking approach provides a comprehensive and adaptive assessment, recognizing that countries may transition between clusters over time.

A detailed analysis of country rankings within each cluster, as reported in Appendix B (Tables B.1–B.3), reveals both inter-cluster transitions and rank shifts within clusters across the three pandemic phases. These shifts can be attributed to multiple factors, including policy adaptations, vaccination strategies, healthcare system resilience, and socio-economic interventions. Such variations have led to a range of outcomes, from highly effective responses to significant challenges in pandemic management. To further support evidence-based policymaking, this study also highlights managerial insights and policy recommendations for improving countries’ preparedness for future health crises. After ranking all clusters, the countries within each cluster and time period were ranked using three MCDM methods: MACONT, COCOSO, and EDAS. The scores obtained from each method were averaged to derive the final rankings. In some cases, the three methods produced consistent rankings, whereas in others, minor variations emerged due to methodological differences. Decision-makers can refer to these rankings to select the most suitable evaluation method based on specific national priorities and use the insights to develop effective strategies for pandemic preparedness and response.

## Discussion

### Findings and interpretation

One key strategy for evaluating countries’ pandemic response performance is identifying the most influential criteria across different phases and clusters. The phase- and cluster-specific importance of each criterion, derived using the CRITIC method, is reported in Appendix C (Tables C.1–C.3). As evident from the tables, the significance of criteria varies across different clusters and phases. While there is a statistically significant correlation between the importance of criteria within clusters across different phases, the importance of criteria differs substantially among clusters. This distinction enhances the accuracy of evaluating countries’ performance within each cluster.

Determining the importance of criteria in each phase, rather than considering their overall significance independent of phase, is crucial for the accuracy and validity of the results. If the criteria importance were assessed holistically without accounting for phase-specific variations, influential criteria in a particular phase could be overlooked, leading to potential misinterpretations and inaccurate rankings. By segmenting the criteria’s importance by phase, this study effectively identifies key performance drivers at each stage of the pandemic, ensuring a more robust ranking of countries and enabling more informed policy decisions tailored to each phase of the crisis. As mentioned in the introduction, this study is the first to integrate Risk INFORM COVID-19 data with existing datasets in the literature. The findings indicate that, across all phases and clusters, the criteria derived from risk data consistently received higher weights than those from previously established criteria in the literature. This finding highlights the critical role of pandemic-specific risk factors in assessing national performance. Notably, criteria related to public health preparedness, socio-economic vulnerability, and healthcare system capacity (key components of the Risk INFORM COVID-19 dataset) ranked consistently higher than traditional indicators. This suggests that conventional evaluation frameworks may overlook essential risk dimensions, underscoring the need to incorporate these dynamic, risk-informed criteria into future pandemic assessment models. This underscores the significant importance of these criteria, which had not previously been considered in the literature, and demonstrates their contribution to improving the accuracy of country performance evaluations.

**Table 2:** Comparison between risk INFORM COVID-19 features importance and literature features importance.

	Average risk INFORM COVID-19 features importance	Average literature features importance
Cluster 1–Phase 1	0.021	0.019
Cluster 2–Phase 1	0.021	0.02
Cluster 3–Phase 1	0.021	0.02
Cluster 1–Phase 2	0.019	0.016
Cluster 2–Phase 2	0.018	0.018
Cluster 3–Phase 2	0.018	0.017
Cluster 1–Phase 3	0.021	0.017
Cluster 2–Phase 3	0.02	0.018
Cluster 3–Phase 3	0.02	0.018

As shown in Table 2, the average weight of the Risk INFORM COVID-19 data criteria across all clusters and phases is higher than that of the existing criteria in the literature. This indicates the significant importance of Risk INFORM COVID-19 data in evaluating country performance and in enhancing the accuracy of performance assessments. A key aspect of this study is examining whether countries experienced improvement, decline, or stability in their performance during the COVID-19 pandemic. This research analyzes these changes from two perspectives:

- Inter-cluster analysis.
- Intra-cluster analysis.

The inter-cluster analysis focuses on countries that transitioned from their initial cluster to a different one during specific phases. Such transitions indicate either an improvement or a decline in a country’s performance within a given period. In contrast, the intra-cluster analysis evaluates changes in countries’ rankings within the same cluster over the three defined time periods, providing a more detailed assessment of performance dynamics within each cluster.

**Table 3:** Inter-cluster analysis of countries.

Country	Phase 1	Phase 2	Phase 3
Bahrain	1	1	3
Belarus	1	1	3
Bulgaria	1	3	1
Kuwait	1	3	3
Malaysia	1	1	3
Oman	1	3	3
Saudi Arabia	1	3	3
Cambodia	2	3	2
Nepal	2	3	2
Cuba	3	1	3
Gabon	3	2	2

Table 3 illustrates the performance trajectories of various countries across three time periods, highlighting their transitions between different clusters. These transitions may reflect fluctuations in national performance across different phases, providing insights into the dynamic nature of country-specific responses over time. The intra-cluster analysis examines the ranking dynamics of countries that remain within the same cluster across the three defined phases of the pandemic. Unlike the inter-cluster transitions, which indicate broader shifts in national performance, intra-cluster variations reflect internal fluctuations driven by differences in healthcare responses, economic resilience, and vaccination strategies. This approach provides a granular assessment of how countries adapted to evolving pandemic conditions while maintaining their relative positioning within their assigned clusters. The analysis of ranking fluctuations within clusters offers valuable insights into the stability of countries’ performance throughout the different phases of the pandemic. Countries in Cluster 1 experienced the greatest ranking fluctuations, with China showing the most significant changes. This suggests that countries in this cluster underwent substantial shifts in their standing due to evolving public health policies, economic adjustments, or changes in healthcare system resilience. In contrast, Cluster 3 exhibited greater rank stability, indicating that countries in this group maintained more consistent performance levels over time. A distinct pattern observed in the ranking dynamics is the relationship with vaccination progress. Countries that implemented mass vaccination programs efficiently tended to exhibit lower fluctuations in ranking, suggesting that rapid immunization was associated with a more stable national response. However, this stability reflects a complex interplay of factors, including healthcare infrastructure, economic resilience, and broader pandemic management strategies, rather than vaccination alone.

It is crucial to acknowledge that while early vaccination rollout coincided with ranking stability, this study relies on observational data and does not establish definitive causal effects. The observed relationship may be influenced by unobserved confounders, such as institutional quality or pre-existing health capacity. While the data indicate that high vaccination coverage aligns with performance stability, establishing a strict causal link would require addressing these confounders through quasi-experimental methods, such as difference-in-differences or synthetic control designs. Consequently, these findings should be interpreted as robust associations that characterize the interplay between vaccination and pandemic resilience, rather than isolated causal impacts. As highlighted in the introduction, vaccination is one of the most crucial strategies for controlling COVID-19. Therefore, equitable vaccine distribution among countries is a critical issue that must be thoroughly examined. In this study, we employ the Gini coefficient to assess the fairness of global vaccine distribution. The Gini coefficient is a widely used statistical measure of inequality, ranging from 0 to 1, where 0 represents perfect equality (all countries receive an equal share of vaccines) and 1 indicates maximum inequality (one country receives all the vaccines while others receive none). By applying this index, we evaluate whether COVID-19 vaccines were distributed fairly worldwide and identify potential disparities in vaccine accessibility.

**Table 4:** Gini coefficient values for vaccination distribution in each cluster.

Cluster	Cluster 1	Cluster 2	Cluster 3	Overall
Gini coefficient	0.124	0.425	0.225	0.347

Table 4 presents the Gini coefficient values for three clusters and the overall dataset, offering insights into the equity of COVID-19 vaccine distribution. The Gini coefficient^55^, a widely used measure of inequality, ranges from 0 (perfect equality) to 1 (maximum inequality), with higher values indicating greater disparities in vaccine allocation.

- Cluster 1 (Gini = 0.124): This cluster exhibits the most equitable vaccine distribution, suggesting minimal disparities among countries in this group. The low Gini coefficient indicates that vaccines were allocated relatively uniformly within this cluster.
- Cluster 2 (Gini = 0.425): This cluster demonstrates the highest level of inequality in vaccine distribution, highlighting substantial disparities in access. The elevated Gini coefficient suggests that some countries in this group received disproportionately higher vaccine supplies than others.
- Cluster 3 (Gini = 0.225): The moderate Gini coefficient in this cluster indicates a certain degree of inequality, though less pronounced than in Cluster 2. While disparities exist, the distribution remains more balanced compared to the most unequal cluster.
- Overall (Gini = 0.347): The overall Gini coefficient suggests considerable inequality in global vaccine distribution.

Despite some clusters showing more equitable allocation patterns, the overall findings highlight significant distributive disparities. However, it is acknowledged that the Gini coefficient, as a scalar metric, primarily captures the statistical outcome of systemic disparities rather than the specific multidimensional causal factors. The observed inequities characterized by an overall Gini of 0.347 and a peak of 0.425 in Cluster 2 are the aggregate result of diverse structural barriers, including supply-chain bottlenecks, intellectual property (IP) regimes, and limited domestic manufacturing capacities. Although the Gini index effectively quantifies the realized gap in vaccine access across national clusters, it does not disaggregate the specific influence of international trade agreements or logistical infrastructure. Consequently, these results should be interpreted as a measure of realized inequity, reflecting the cumulative impact of these underlying multidimensional constraints on global health outcomes. Addressing these imbalances requires strategic interventions and international collaboration that target these root structural drivers rather than merely addressing the statistical symptoms.

### Sensitivity analysis

#### Stability with respect to clustering algorithms

To assess the robustness of the proposed clustering–ranking framework, a sensitivity analysis was conducted by comparing the country rankings obtained from two different clustering techniques: K-means and Gaussian Mixture Models (GMM). The objective of this analysis is to evaluate whether the final country rankings are sensitive to the choice of clustering algorithm or remain stable under methodological variations. The analysis was performed independently for all three pandemic phases, namely the pre-vaccination, vaccination, and post-vaccination phases. For each phase, clustering was carried out using both K-means and GMM with the same number of clusters, and the resulting country rankings were compared using the Spearman rank correlation coefficient. This measure captures the degree of monotonic agreement between the two ranking lists and is widely used to assess ranking robustness. The results indicate a high level of agreement between the rankings produced by K-means and GMM across all phases. Specifically, the Spearman correlation coefficients are 0.979 for the pre-vaccination phase, 0.918 for the vaccination phase, and 0.986 for the post-vaccination phase. These values demonstrate a strong positive correlation, suggesting that the relative ordering of countries remains largely unchanged despite variations in the clustering methodology. Notably, the slightly lower correlation observed during the vaccination phase can be attributed to the dynamic and transitional nature of this period, during which countries experienced heterogeneous vaccination rollout speeds and policy responses. Nevertheless, the correlation remains well above commonly accepted robustness thresholds, confirming the stability of the proposed framework even under more volatile conditions. Overall, the consistency of ranking outcomes across different clustering approaches and pandemic phases confirms that the proposed methodology is robust, reliable, and not overly sensitive to the choice of clustering algorithm. This strengthens the validity of the study’s conclusions and supports the use of the proposed framework for comparative country-level analysis in different pandemic stages.

#### Robustness of country rankings under data perturbations

To rigorously assess the robustness of the proposed MCDM framework, we performed a comprehensive sensitivity analysis across all pandemic phases: pre-vaccination, vaccination, and post-vaccination. For this purpose, small perturbations were introduced to the numerical input criteria to simulate the potential variability inherent in real-world datasets. Specifically, we applied a ± 5% random fluctuation to each numerical feature, ensuring that the perturbations were minor and within realistic bounds, while preserving the original distribution of the data. The complete MCDM pipeline, including normalization, CRITIC weighting, and aggregation through MACONT, COCOSO, and EDAS, was re-executed on the perturbed datasets for all clusters and phases. The resulting rankings were then quantitatively compared to the original rankings using the Spearman rank correlation coefficient, which measures the monotonic agreement between two ranking lists (Table 5).

**Table 5:** Quantitative assessment of ranking stability across clusters and pandemic phases.

Cluster	Phase	Avg Spearman	Std Dev
1	Pre-vaccination	0.979	0.004
2	Pre-vaccination	0.988	0.002
3	Pre-vaccination	0.959	0.004
1	Vaccination	0.958	0.010
2	Vaccination	0.994	0.001
3	Vaccination	0.967	0.003
1	Post-vaccination	0.980	0.003
2	Post-vaccination	0.993	0.001
3	Post-vaccination	0.960	0.003

Table 1 presents the results of this analysis. For each cluster and phase, the average Spearman correlation and its standard deviation across multiple perturbation trials are reported. The consistently high correlation values (all above 0.95) indicate that the relative ordering of countries remains largely unchanged despite small variations in input data. This demonstrates that the proposed framework is stable and not sensitive to minor fluctuations or noise in the input criteria. The low standard deviations further confirm the reliability and reproducibility of the rankings, underscoring the robustness of the methodology across different pandemic phases.

Overall, these findings provide strong evidence that the proposed MCDM framework produces reliable, resilient, and interpretable country rankings, suitable for comparative policy analysis and decision support, even in the presence of minor data uncertainties.

### Managerial insight

The findings of this study provide crucial insights for policymakers and decision-makers in managing future public health crises. The implications extend to strategic planning, resource allocation, and policy optimization. Key managerial perspectives derived from this research include:

- Strategic planning for crisis management: By analyzing the performance of countries during the COVID-19 pandemic, policymakers can develop more adaptive and resilient response frameworks for future health crises. This study highlights effective governance models that enhance crisis preparedness.
- Evidence-based resource allocation: The integration of MCDM methods allows decision-makers to prioritize resource distribution based on empirical data rather than heuristic approaches. This ensures efficient use of medical supplies, financial aid, and vaccination programs, particularly in vulnerable regions.
- Optimization of public health policies: Understanding the critical factors influencing national performance enables governments to design more effective and proactive public health strategies. The application of the CRITIC method ensures that decision-making is based on objective, weighted criteria, thereby reducing inefficiencies in policy implementation.
- Enhancing international collaboration: The study’s insights facilitate better coordination among nations, encouraging the adoption of best practices and the establishment of global health partnerships. By identifying successful national strategies, countries can benchmark their policies against top-performing nations and enhance collective pandemic resilience.

Beyond immediate crisis response, the proposed framework can be institutionalized as a continuous monitoring and evaluation tool within national health governance systems. By periodically updating indicators and reapplying the clustering–ranking mechanism, decision-makers can track shifts in national resilience over time and detect early signals of systemic stress before they escalate into full-scale crises. This enables a transition from reactive to anticipatory governance, where corrective actions—such as targeted capacity building, regulatory adjustments, or contingency planning—can be implemented proactively. Furthermore, the modular structure of the framework allows it to be adapted to other large-scale disruptions, including future pandemics, climate-related health shocks, or supply-chain-induced healthcare emergencies, thereby increasing its long-term managerial value beyond COVID-19. This research underscores the importance of data-driven decision-making in public health management. It provides policymakers with a structured approach to enhance preparedness, efficiency, and collaboration in global health governance.

## Conclusion

This study presents a comprehensive, data-driven framework integrating Multi-Criteria Decision-Making (MCDM) techniques with Machine Learning (ML) to evaluate the effectiveness of pandemic responses across 143 countries systematically. Unlike previous research that primarily focuses on static assessments, this study introduces a vaccination-dependent temporal framework that enables dynamic evaluation across the pre-vaccination, vaccination, and post-vaccination phases. The integration of the Risk INFORM COVID-19 dataset with conventional indicators marks a significant methodological advancement, enabling a more holistic, risk-informed assessment of national pandemic responses.

The clustering analysis identified three distinct country groups, each exhibiting unique structural and policy-driven characteristics. The transitions between clusters revealed the critical influence of early vaccination efforts, healthcare infrastructure, socio-economic resilience, and public policy stringency in shaping pandemic outcomes. Additionally, intra-cluster ranking dynamics showed that countries with proactive and adaptive public health strategies maintained greater stability, underscoring the importance of data-driven, flexible policymaking in crisis management.

Despite the robustness of the proposed framework, specific inherent challenges should be acknowledged. While our equity assessment uses the Gini coefficient to capture relative disparities in vaccine distribution, complementary structural factors, such as intellectual property frameworks and cold-chain capacity were conceptually recognized but intentionally excluded from the quantitative scope due to global data inconsistencies. Their acknowledgment strengthens the contextual interpretation of our equity findings without altering the robustness of the empirical results. Furthermore, while the definition of pandemic phases relies on global epidemiological milestones to ensure temporal consistency, this ‘global clock’ approach warrants cautious interpretation. By standardizing the start of the ‘Vaccination Phase’ based on the first global administration (in the UK), the framework may inadvertently penalize ‘late-starter’ nations—primarily developing countries that faced significant delays in vaccine access due to global supply chain inequities. Consequently, lower rankings for these nations during the vaccination phase may reflect structural barriers to access rather than deficiencies in internal management efficiency. Readers should, therefore, distinguish between performance metrics driven by logistical readiness and those constrained by exogenous supply shortages. Another critical limitation stems from the data exclusion criteria. Although the initial dataset covered 231 countries, the reduction to 143 nations driven by the need to maintain statistical reliability introduces unavoidable selection bias. The excluded nations are disproportionately low-income or fragile states characterized by weaker health information infrastructure. Consequently, the study’s findings regarding global equity and resilience likely underestimate the true depth of disparities, as the most resource-deprived nations are underrepresented in the analysis. Therefore, the conclusions should be interpreted as a conservative baseline, with the understanding that the unobserved reality in data-poor regions is likely more severe than reported. Future studies could mitigate this by incorporating qualitative assessments or proxy indicators for data-scarce regions.

The hybrid MCDM approach, integrating MACONT, COCOSO, and EDAS, enabled a multi-faceted and robust ranking of national performances. The comparative results indicate that while certain countries demonstrated consistently high performance, others experienced substantial ranking fluctuations, primarily influenced by the effectiveness of policy interventions and vaccine accessibility. To further explore vaccine equity, this study used the Gini coefficient, which revealed significant disparities, particularly in low-income countries. These findings underscore the urgent need for more equitable vaccine distribution frameworks to mitigate inequalities in future global health crises.

The findings of this study offer several key policy implications. First, countries that prioritized early vaccination and stringent public health measures demonstrated greater stability and faster recovery, reinforcing the need to maintain agile, adaptive healthcare policies. Second, observed inequalities in vaccine distribution highlight the imperative of international cooperation and equitable allocation mechanisms to ensure a more just global response. Third, the importance of dynamic, real-time decision-making suggests that integrating MCDM and ML-based monitoring systems can provide policymakers with timely, evidence-driven insights to enhance crisis preparedness.

For future research, expanding the dataset to include long-term recovery indicators will provide deeper insights into the economic and social repercussions of pandemic management strategies. This study does not differentiate between vaccine types, procurement mechanisms, or booster-specific uptake, as the analysis focuses on aggregated national vaccination coverage; future research may incorporate vaccine efficacy heterogeneity and supply-chain characteristics to provide a more granular assessment. Additionally, incorporating Deep Learning models alongside MCDM techniques can further enhance the predictive and adaptive capabilities of decision-support systems, enabling more proactive and resilient crisis response planning.

By leveraging advanced analytical frameworks, policymakers can refine global health strategies, strengthen preparedness for future crises, and improve the resilience of healthcare systems, ensuring a more effective, equitable, and data-driven response to future pandemics.

## Supplementary Information

Below is the link to the electronic supplementary material.

Supplementary Material 1

## Data Availability

All data supporting the findings of this study are either publicly available from third-party repositories cited in the Methods section. Where permissible, the analysis-ready dataset and accompanying data dictionary used to generate the figures and tables are provided alongside the code at the repository listed below. Hyperlinks and persistent identifiers for all third-party sources are provided in-text.All analysis scripts (data preprocessing, imputation, clustering, MCDM computations, robustness checks, and figure generation) are openly available at Figshare: (10.6084/m9.figshare.30174001) .Supplementary data associated with this article can be found in the online version at: Demographical and Risk INFORM COVID-19 Dataset (https://drmkc.jrc.ec.europa.eu/inform-index/portals/0/InfoRM/Covid19/INFORM%20COVID-19%20RISK%20INDEX%20v014.xlsx) Vaccination Dataset (https://srhdpeuwpubsa.blob.core.windows.net/whdh/COVID/vaccination-data.csv) Time-Series Dataset (https://github.com/15e70f54-3883-4e52-9c02-b9a212ec264b).

## References

[1] Yang, L. et al. COVID-19: Immunopathogenesis and immunotherapeutics. *Signal. Transduct. Target. Therapy*. **5** (1), 128 (2020). 10.1038/s41392-020-00243-2 PMC7381863 32712629

[2] Khanduzi, R. et al. A novel collocation method with a coronavirus optimization algorithm for the optimal control of COVID-19: a case study of Wuhan, China. *Comput. Biol. Med.* **178**, 108680 (2024). 38843571 10.1016/j.compbiomed.2024.108680

[3] Velavan, T. P. & Meyer, C. G. The COVID-19 epidemic. *Tropical Med. Int. Health*. **25** (3), 278 (2020). 10.1111/tmi.13383 PMC7169770 32052514

[4] Clemente-Suárez, V. J. et al. The impact of the COVID-19 pandemic on social, health, and economy. *Sustainability* **13** (11), 6314 (2021).

[5] Rezapour Niari, M., Eshghi, K., Fatahi, O. & Valilai Adaptive capacity management in cloud manufacturing hyper-network platform: case of COVID-19 equipment production. *Int. J. Manag. Sci. Eng. Manag.* **17** (4), 239–258 (2022).

[6] Worldometer *COVID-19 Coronavirus Pandemic*. (2025).

[7] Tollefson, J. COVID curbed 2020 carbon emissions-but not by much. *Nature* **589** (343), 373–377 (2021). 10.1038/d41586-021-00090-3 33452515

[8] Rezapour Niari, M., Eshgi, K., Fatahi, O. & Valilai Topology analysis of manufacturing service supply–demand hyper-network considering QoS properties in the cloud manufacturing system. *Robot. Comput. Integr. Manuf.* **72**, 102205 (2021).

[9] Koirala, A. et al. Vaccines for COVID-19: the current state of play. *Paediatr. Respir. Rev.* **35**, 43–49 (2020). 32653463 10.1016/j.prrv.2020.06.010 PMC7301825

[10] Excler, J. L. et al. Vaccine development for emerging infectious diseases. *Nat. Med.* **27** (4), 591–600 (2021). 33846611 10.1038/s41591-021-01301-0

[11] Jung, J. Preparing for the coronavirus disease (COVID-19) vaccination: evidence, plans, and implications. *J. Korean Med. Sci.*, **36**(7). (2021). 10.3346/jkms.2021.36.e59 PMC7900528 33619920

[12] Morens, D. M., Folkers, G. K. & Fauci, A. S. The concept of classical herd immunity May not apply to COVID-19. *J. Infect. Dis.* **226** (2), 195–198 (2022). 35356987 10.1093/infdis/jiac109 PMC9129114

[13] Dzinamarira, T. et al. Unpacking the implications of SARS-CoV-2 breakthrough infections on COVID-19 vaccination programs. *Vaccines* **10** (2), 252 (2022). 35214710 10.3390/vaccines10020252 PMC8879800

[14] Kelly, S. L. et al. COVID-19 vaccine booster strategies in light of emerging viral variants: frequency, timing, and target groups. *Infect. Dis. Therapy*. **11** (5), 2045–2061 (2022). 10.1007/s40121-022-00683-z PMC9464609 36094720

[15] Haug, N. et al. Ranking the effectiveness of worldwide COVID-19 government interventions. *Nat. Hum. Behav.* **4** (12), 1303–1312 (2020). 33199859 10.1038/s41562-020-01009-0

[16] Yao, L. et al. Variations of COVID-19 mortality are affected by economic disparities across countries. *Sci. Total Environ.* **832**, 154770 (2022). 35341873 10.1016/j.scitotenv.2022.154770 PMC8949690

[17] Calabrò, G. E. et al. The impact of vaccination on COVID-19 burden of disease in the adult and elderly population: a systematic review of Italian evidence. *Vaccines* **11** (5), 1011 (2023). 37243115 10.3390/vaccines11051011 PMC10222140

[18] Garai, T. & Garg, H. An interpreter ranking index-based MCDM technique for COVID-19 treatments under a bipolar fuzzy environment. *Results Control Optim.* **12**, 100242 (2023).

[19] Coccia, M. Preparedness of countries to face COVID-19 pandemic crisis: strategic positioning and factors supporting effective strategies of prevention of pandemic threats. *Environ. Res.* **203**, 111678 (2022). 34280421 10.1016/j.envres.2021.111678 PMC8284056

[20] Albahri, O. et al. Novel dynamic fuzzy decision-making framework for COVID-19 vaccine dose recipients. *J. Adv. Res.* **37**, 147–168 (2022). 35475277 10.1016/j.jare.2021.08.009 PMC8378994

[21] Sarwar, A. et al. Measuring vaccination willingness in response to COVID-19 using a multi-criteria-decision making method. *Hum. Vaccines Immunother*. **17** (12), 4865–4872 (2021). 10.1080/21645515.2021.2004836 PMC8903990 34856879

[22] Alemdar, K. D. et al. Accessibility of vaccination centers in COVID-19 outbreak control: A gis-based multi-criteria decision making approach. *ISPRS Int. J. Geo-Inf*. **10** (10), 708 (2021).

[23] Seker, S. et al. Risk assessment approach for analyzing risk factors to overcome pandemic using interval-valued q-rung orthopair fuzzy decision making method. *Appl. Soft Comput.* **132**, 109891 (2023). 36471784 10.1016/j.asoc.2022.109891 PMC9714129

[24] Makki, A. A. & Alqahtani, A. Y. Capturing the effect of the COVID-19 pandemic outbreak on the financial performance disparities in the energy sector: A hybrid MCDM-Based evaluation approach. *Economies* **11** (2), 61 (2023).

[25] Ergülen, A. & Çalık, A. F-BWM-MARCOS approach for performance evaluation of Türkiye’s top 500 industrial enterprises in the pre-pandemic and pandemic era. *Int. J.* (2024).

[26] Ros, F. et al. *Addressing the Covid-19 Pandemic and Future Public Health Challenges Through Global Collaboration and a data-driven Systems Approach* (Wiley Online Library, 2021). 10.1002/lrh2.10253 PMC7744897 33349796

[27] Tao, Y. et al. A survey on data-driven covid-19 and future pandemic management. *ACM Comput. Surveys*. **55** (7), 1–36 (2022).

[28] Ibrahim, Z., Tulay, P. & Abdullahi, J. Multi-region machine learning-based novel ensemble approaches for predicting COVID-19 pandemic in Africa. *Environ. Sci. Pollut. Res.* **30** (2), 3621–3643 (2023). 10.1007/s11356-022-22373-6 PMC9365685 35948797

[29] Khattab, R., Abdelmaksoud, I. R. & Abdelrazek, S. Automated detection of COVID-19 and pneumonia diseases using data mining and transfer learning algorithms with focal loss from chest X-ray images. *Appl. Soft Comput.* **162**, 111806 (2024).

[30] Sotoudeh-Anvari, A. The applications of MCDM methods in COVID-19 pandemic: A state of the Art review. *Appl. Soft Comput.* **126**, 109238 (2022). 35795407 10.1016/j.asoc.2022.109238 PMC9245376

[31] Moosazadeh, M. et al. A machine learning-driven spatio-temporal vulnerability appraisal based on socio-economic data for COVID-19 impact prevention in the US counties. *Sustain. Cities Soc.* **83**, 103990 (2022). 35692599 10.1016/j.scs.2022.103990 PMC9167466

[32] Zgheib, R. et al. Towards an ML-based semantic IoT for pandemic management: A survey of enabling technologies for COVID-19. *Neurocomputing* **528**, 160–177 (2023). 36647510 10.1016/j.neucom.2023.01.007 PMC9833856

[33] Fu, C. et al. Data-driven decision making based on evidential reasoning approach and machine learning algorithms. *Appl. Soft Comput.* **110**, 107622 (2021).

[34] Khan, M. et al. Applications of artificial intelligence in COVID-19 pandemic: A comprehensive review. *Expert Syst. Appl.* **185**, 115695 (2021). 34400854 10.1016/j.eswa.2021.115695 PMC8359727

[35] Hadi, M. U. et al. A lightweight CORONA-NET for COVID-19 detection in X-ray images. *Expert Syst. Appl.* **225**, 120023 (2023). 37063778 10.1016/j.eswa.2023.120023 PMC10088342

[36] Chakraborty, S. & Mali, K. SUFEMO: a superpixel based fuzzy image segmentation method for COVID-19 radiological image Elucidation. *Appl. Soft Comput.* **129**, 109625 (2022). 36124000 10.1016/j.asoc.2022.109625 PMC9474408

[37] Cheong, Q. et al. Predictive modeling of vaccination uptake in US counties: A machine learning–based approach. *J. Med. Internet. Res.* **23** (11), e33231 (2021). 34751650 10.2196/33231 PMC8623305

[38] Ong, E. et al. COVID-19 coronavirus vaccine design using reverse vaccinology and machine learning. *Front. Immunol.* **11**, 1581 (2020). 32719684 10.3389/fimmu.2020.01581 PMC7350702

[39] Alkan, N. & Kahraman, C. Evaluation of government strategies against COVID-19 pandemic using q-rung orthopair fuzzy TOPSIS method. *Appl. Soft Comput.* **110**, 107653 (2021). 34226821 10.1016/j.asoc.2021.107653 PMC8241659

[40] Ecer, F. & Pamucar, D. MARCOS technique under intuitionistic fuzzy environment for determining the COVID-19 pandemic performance of insurance companies in terms of healthcare services. *Appl. Soft Comput.* **104**, 107199 (2021). 34720778 10.1016/j.asoc.2021.107199 PMC8546419

[41] Hezam, I. M. et al. COVID-19 vaccine: A neutrosophic MCDM approach for determining the priority groups. *Results Phys.* **20**, 103654 (2021). 33520620 10.1016/j.rinp.2020.103654 PMC7832528

[42] Shanker, S. et al. Enhancing resiliency of perishable product supply chains in the context of the COVID-19 outbreak. *Int. J. Logistics Res. Appl.* **25** (9), 1219–1243 (2022).

[43] Salehi, V. et al. An MCDM approach to assessing influential factors on healthcare providers’ safe performance during the COVID-19 pandemic: probing into demographic variables. *J. Saf. Sci. Resil.* **4** (3), 274–283 (2023). 40476963 10.1016/j.jnlssr.2023.05.002 PMC10249363

[44] Ahmad, S. et al. Analysing the impact of COVID-19 pandemic on the psychological health of people using fuzzy MCDM methods. *Oper. Res. Perspect.* **10**, 100263 (2023).

[45] Özgür, İ. & GÜRE, M. D. P. Evaluation of rare diseases policy performance of Oecd countries using Mcdm methods. *Health Policy Technol.* **10** (3), 100537 (2021).

[46] Matsumoto, K., Makridou, G. & Doumpos, M. Evaluating environmental performance using data envelopment analysis: the case of European countries. *J. Clean. Prod.* **272**, 122637 (2020).

[47] Mete, S. et al. An integrated hybrid MCDM approach to evaluate countries’ COVID-19 risks. *Socio-Economic Plann. Sci.* **90**, 101744 (2023).

[48] Mohammed, M. A. et al. Benchmarking methodology for selection of optimal COVID-19 diagnostic model based on entropy and TOPSIS methods. *IEEE Access.* **8**, 99115–99131 (2020).

[49] Aggarwal, L., Goswami, P. & Sachdeva, S. Multi-criterion intelligent decision support system for COVID-19. *Appl. Soft Comput.* **101**, 107056 (2021). 33390874 10.1016/j.asoc.2020.107056 PMC7771316

[50] Chowdhury, N. K. et al. Machine learning for detecting COVID-19 from cough sounds: an ensemble-based MCDM method. *Comput. Biol. Med.* **145**, 105405 (2022). 35318171 10.1016/j.compbiomed.2022.105405 PMC8926945

[51] Aydin, N. & Yurdakul, G. Assessing countries’ performances against COVID-19 via WSIDEA and machine learning algorithms. *Appl. Soft Comput.* **97**, 106792 (2020). 33071686 10.1016/j.asoc.2020.106792 PMC7556230

[52] Van Buuren, S. Groothuis-Oudshoorn, mice: multivariate imputation by chained equations in R. *J. Stat. Softw.* **45**, 1–67 (2011).

[53] Alin, A. Multicollinearity. *Wiley Interdiscipl. Rev. Comput. Stat.* **2** (3), 370–374 (2010).

[54] Tsagris, M. & Pandis, N. Multicollinearity. *Am. J. Orthod. Dentofac. Orthop.* **159**(5): 695–696. (2021). 10.1016/j.ajodo.2021.02.005 33931224

[55] Gutjahr, W. J. Fair and efficient vaccine allocation: A generalized Gini index approach. *Prod. Oper. Manage.* **32** (12), 4114–4134 (2023).

[56] MacQueen, J. Some methods for classification and analysis of multivariate observations. In *Proceedings of the Fifth Berkeley Symposium on Mathematical Statistics and Probability, Volume 1: Statistics* (University of California Press, 1967).

[57] Diakoulaki, D., Mavrotas, G. & Papayannakis, L. Determining objective weights in multiple criteria problems: the critic method. *Comput. Oper. Res.* **22** (7), 763–770 (1995).

[58] Wen, Z., Liao, H. & Zavadskas, E. K. MACONT: mixed aggregation by comprehensive normalization technique for multi-criteria analysis. *Informatica* **31** (4), 857–880 (2020).

[59] Yazdani, M. et al. A combined compromise solution (CoCoSo) method for multi-criteria decision-making problems. *Manag. Decis.* **57** (9), 2501–2519 (2019).

[60] Keshavarz Ghorabaee, M. et al. Multi-criteria inventory classification using a new method of evaluation based on distance from average solution (EDAS). *Informatica* **26** (3), 435–451 (2015).

