# Supplementary material for: A multi-phase, multi-method assessment of national COVID-19 vaccination performance with equity analysis
Source: Sci Rep. 2026 Feb 10;16:8140. doi: 10.1038/s41598-026-39677-z (PMC12960841; doi:10.1038/s41598-026-39677-z)
Supplement: Supplementary file 1 — Supplementary Material 1 [file 41598_2026_39677_MOESM1_ESM.docx]

**Appendix A. Methodological Details**

This appendix provides the mathematical formulations and algorithmic steps of the K-means clustering and the MCDM methods used in this study.

**A.1. K-means Clustering**

# K-means [57] clustering is a widely adopted unsupervised learning technique for partitioning data into distinct, non-overlapping groups based on inherent similarities. This method is particularly valued for its computational efficiency, scalability, and ease of interpretation, making it one of the most frequently utilized clustering algorithms in data analysis. The K-means algorithm operates iteratively, beginning with initialization, where the number of clusters (K) is predefined, and K initial centroids are selected—often randomly—from the dataset. In the assignment step, each data point is allocated to the cluster with the nearest centroid, typically determined using Euclidean distance, ensuring that observations within the same cluster exhibit maximum similarity. The update step then recalculates the centroids by computing the mean of all assigned data points within each cluster. This process iterates until convergence is achieved, signifying that changes in centroid positions become negligible or a predefined stopping criterion is met. Despite its simplicity, K-means remains a powerful tool for identifying hidden structures within data, enabling meaningful pattern recognition, segmentation, and exploratory analysis across a wide range of disciplines. However, its performance is influenced by factors such as the choice of K, sensitivity to initial centroid placement, and the presence of outliers, necessitating careful preprocessing and parameter selection.

**A.2.** **Multi-Criteria Decision-Making**

One common approach to evaluating the performance of a group of alternatives, such as countries in the context of pandemic management, is to employ **MCDM methodologies**. In line with the existing literature, this study first applies the **CRITIC method** to determine the **weights of the criteria** across three **vaccination-dependent phases**. Following this, the **MACONT, COCOSO, and EDAS methods** are utilized to assess and rank countries **within each cluster** as well as to evaluate the overall performance of the clusters. The combination of these three MCDM methods ensures a robust, multidimensional evaluation. MACONT applies comprehensive normalization to eliminate bias in ranking, COCOSO integrates both additive and multiplicative models to enhance ranking stability, and EDAS considers both positive and negative deviations from the average, making it well-suited for handling uncertainty in performance assessment.

To obtain a unified and robust final ranking, this study aggregates the results of MACONT, COCOSO, and EDAS using a simple arithmetic averaging approach. This strategy follows the principles of rank aggregation and ensemble decision-making, where combining multiple independent MCDM methods reduces the dominance of any single method and improves the stability of the final ranking. The three selected methods represent distinct families of MCDM models—comprehensive normalization (MACONT), hybrid additive–multiplicative aggregation (COCOSO), and deviation-based evaluation (EDAS). Because these methods rely on different normalization schemes and decision philosophies, averaging their ranked outputs provides a theoretically justified compromise solution that reflects consensus across heterogeneous decision rules. This ensemble-style aggregation has been widely recommended in the MCDM literature to enhance robustness, mitigate methodological bias, and improve the reliability of final rankings.

This multi-method approach provides a well-rounded perspective on national pandemic responses, reducing the potential bias of any single decision-making technique. This integrated approach ensures a **comprehensive and systematic evaluation** of national responses to the COVID-19 pandemic.

A.2.1. CRITIC Method

# To evaluate the relative importance of the criteria in this study, we employ the CRITIC method—a robust weighting approach that leverages both the degree of contrast (via standard deviation) and the interdependencies (via correlation coefficients) among criteria [58]. This method is particularly advantageous because it quantifies not only the variability of each attribute but also the extent to which its attributes conflict or agree with one another, thus avoiding the often unrealistic assumption of attribute independence. In the initial step, we define the decision matrix $X= \left[ x_{ij} \right]_{m\times n}$, where $i\in\left\{ 1.2\ldots.m \right\}$ and $j\in\left\{ 1.2\ldots.n \right\}$. Subsequently, the matrix is normalized to ensure that all entries fall within the interval $[0. 1]$. For benefit criteria, the normalization is performed as Eq $(1)$:

$$r_{ij}= \frac{x_{ij}-x_{j}^{min}}{x_{j}^{max}-x_{j}^{min}} \forall i\in\left\{ 1.2\ldots.m \right\}, j\in\left\{ 1.2\ldots.n \right\} (1)$$

while for cost criteria, the normalization is given by Eq $(2)$:

$$r_{ij}= \frac{x_{j}^{max}-x_{ij}}{x_{j}^{max}-x_{j}^{min}} \forall i\in\left\{ 1.2\ldots.m \right\}, j\in\left\{ 1.2\ldots.n \right\} (2)$$

In the subsequent step, the amount of information $Cj$extracted from criterion $j$ is determined using its standard deviation $\sigma j$ along with the normalized values:

$$C_{j}= \sigma j\sum_{i=1}^{m} 1-r_{ij} \forall j\in\left\{ 1.2\ldots.n \right\} (3)$$

# Finally, the weight of each criterion is derived by normalizing these information measures:

$$w_{j}=\frac{C_{j}}{\sum_{j=1}^{n} C_{j}} (4)$$

# This systematic procedure not only enhances the objectivity of the weighting process but also facilitates the integration of qualitative attributes into a quantitative framework, thereby strengthening the overall analysis.

Although CRITIC relies on statistical dispersion, potential overweighting of noisy indicators was mitigated through prior preprocessing, including standardization, outlier control, and correlation screening. In addition, the criteria set was defined based on established epidemiological frameworks, ensuring that domain-relevant indicators are embedded before objective weighting is applied. Consequently, CRITIC serves as a complementary, data-driven mechanism that enhances, rather than replaces, the role of public-health expertise in the weighting process.

*A.2.2. MACONT Decision-Making Technique*

The Mixed Aggregation by Comprehensive Normalization Technique (MACONT) [59] is a robust MCDM method that evaluates alternatives by integrating comprehensive normalization and mixed aggregation techniques. This method ensures an unbiased comparison by transforming all criteria into a unified scale while effectively handling both benefit and cost criteria. The method requires three key inputs:

1. Criterion weights, obtained using the CRITIC method,
2. Criterion categories (benefit or cost),
3. Decision matrix.

The decision matrix is structured as follows:

$$X= \left[ x_{ij} \right]_{m\times n} \forall i\in\left\{ 1.2\ldots.m \right\}, j\in\left\{ 1.2\ldots.n \right\} (5)$$

where $x_{ij}$ represents the performance value of the alternative $i$ under criterion $j$.

To ensure comparability across different measurement units, a **min-max normalization approach** is applied.

For benefit criteria, normalization is performed as follows:

$$r_{ij}= \frac{x_{ij}-x_{j}^{min}}{x_{j}^{max}-x_{j}^{min}} \forall i\in\left\{ 1.2\ldots.m \right\}, j\in\left\{ 1.2\ldots.n \right\} (6)$$

For cost criteria, the normalization is performed as follows:

$$r_{ij}= \frac{x_{j}^{max}-x_{ij}}{x_{j}^{max}-x_{j}^{min}} \forall i\in\left\{ 1.2\ldots.m \right\}, j\in\left\{ 1.2\ldots.n \right\} (7)$$

where $max(x_{j})$and $min(x_{j})$denote the maximum and minimum values for the criterion $j$, respectively, this transformation maps all values onto a $[0.1]$ scale, ensuring consistency in the evaluation process.

Each criterion is assigned a weight $w_{j}$, determined using the CRITIC method. The weighted normalized values are then computed as:

$$v_{ij}=r_{ij}\times w_{j} (8)$$

Where $v_{ij}$ represents the weighted normalized score for the alternative $i$ under criterion $j$.

The final score for each alternative is derived using:

$$S_{i}=\sum_{j=1}^{n} v_{ij} (9)$$

*B.2.3. COCOSO*

This method integrates the simple Weighted Sum Method (WSM) and the Weighted Product Method (WPM). The process begins by normalizing the decision matrix, as denoted in Eqs. $(10)$and $(11)$ [60]:

$$X= \left[ x_{ij} \right]_{m\times n} \forall i\in\left\{ 1.2\ldots.m \right\}, j\in\left\{ 1.2\ldots.n \right\} (10)$$

For benefit criteria, normalization is performed as follows:

$$r_{ij}= \frac{x_{ij}-x_{j}^{min}}{x_{j}^{max}-x_{j}^{min}} \forall i\in\left\{ 1.2\ldots.m \right\}, j\in\left\{ 1.2\ldots.n \right\} (11)$$

For cost criteria, the normalization is performed as follows:

$$r_{ij}= \frac{x_{j}^{max}-x_{ij}}{x_{j}^{max}-x_{j}^{min}} \forall i\in\left\{ 1.2\ldots.m \right\}, j\in\left\{ 1.2\ldots.n \right\} (12)$$

Following normalization, the weighted sum ($S_{i} )$ and the weighted product $(P_{i})$ e computed for each alternative using the CRITIC-derived weights $(w_{j})$:

$$S_{i}=\sum_{j=1}^{n} \left( w_{j}r_{ij} \right) \forall i\in\left\{ 1.2\ldots.m \right\} (13)$$

$$P_{i}=\sum_{j=1}^{n} {r_{ij}}^{w_{j}} \forall i\in\left\{ 1.2\ldots.m \right\} (14)$$

Based on these values, three composite scores are formulated:

$$k_{ia}=\frac{P_{i}+S_{i}}{\sum_{i=1}^{m} \left( P_{i}+S_{i} \right)} (15)$$

$$k_{ib}=\frac{S_{i}}{min S_{i}}+\frac{P_{i}}{min P_{i}} (16)$$

$$k_{ic}=\frac{\lambda\left( S_{i} \right)+\left( 1-\lambda\right)\left( P_{i} \right)}{\lambda\max S_{i}+\left( 1-\lambda\right)\max P_{i}} \left( 0\leq\lambda\leq1 \right) (17)$$

Here,$k_{ia}$ represents the arithmetic mean of the WSM and WPM scores, while $k_{ib}$ expresses their relative performance compared to the best alternative. The parameter $k_{ic}$ provides a compromise between $WSM$ and $WPM$, with$\lambda$ determined by the decision maker (DM), typically set to 0.5 for balanced weighting.

The final ranking score ($k_{i}$) is computed by integrating these three measures:

$$k_{i}={(k_{ia}k_{ib}k_{ic})}^{\frac{1}{3}}+\frac{1}{3}\left( k_{ia}+k_{ib}+k_{ic} \right) (18)$$

The alternative with the highest$k_{i}$ value is identified as the most favorable choice. This approach ensures a balanced and robust ranking by leveraging both additive and multiplicative decision-making principles.

*A.2.4. EDAS*

Another alternative prioritization method utilized in this study, in alignment with the literature, is the Evaluation based on Distance from Average Solution (EDAS) [61]. This method requires three key inputs:

1. Criterion weights obtained using the CRITIC method,
2. Criterion categories (benefit or cost),
3. Decision matrix.

The decision matrix is first established as follows:

$$X= \left[ x_{ij} \right]_{m\times n} \forall i\in\left\{ 1.2\ldots.m \right\}, j\in\left\{ 1.2\ldots.n \right\} (19)$$

The mean value of each criterion across all alternatives is computed using:

$${AV}_{j}=\frac{\sum_{i=1}^{n} x_{ij}}{n} (20)$$

which is represented as a row vector:

$$\left[ {AV}_{j} \right]_{1\times m} (21)$$

In the next step, using the Positive Distance from Average $(PDA)$ and Negative Distance from Average $(NDA)$ equations, we calculated the positive and negative separation from the mean based on criterion categories as defined in Eqs. $(19)$ and $(20)$:

$$\text{Benefit criterion:}\left\{ \begin{aligned} {NDA}_{ij}=\frac{max\left\{ 0.\left( {AV}_{j}-x_{ij} \right) \right\}}{{AV}_{j}} \\ {PDA}_{ij}=\frac{max\left\{ 0.\left( {x_{ij}-AV}_{j} \right) \right\}}{{AV}_{j}} \end{aligned} \right\} (22)$$

$$\text{Cost criterion:}\left\{ \begin{aligned} {PDA}_{ij}=\frac{max\left\{ 0.\left( {AV}_{j}-x_{ij} \right) \right\}}{{AV}_{j}} \\ {NDA}_{ij}=\frac{max\left\{ 0.\left( {x_{ij}-AV}_{j} \right) \right\}}{{AV}_{j}} \end{aligned} \right\} (23)$$

The $\mathrm{PDA}$ and $\mathrm{NDA}$ values are then weighted by their respective criterion weights $(w_{j})$to obtain the weighted sums ${SP}_{i}$ and ${SN}_{i}$ for each alternative:

$${SP}_{i}=\sum_{j=1}^{m} w_{j}{PDA}_{ij} \forall i\in\left\{ 1.2\ldots.m \right\} (24)$$

$${SN}_{i}=\sum_{j=1}^{m} w_{j}{NDA}_{ij} \forall i\in\left\{ 1.2\ldots.m \right\} (25)$$

The obtained values are then normalized as follows:

$${NSP}_{i}=\frac{{SP}_{i}}{max({SP}_{i})} \forall i\in\left\{ 1.2\ldots.m \right\} (26)$$

$${NSN}_{i}=1-\frac{{SN}_{i}}{max({SN}_{i})} \forall i\in\left\{ 1.2\ldots.m \right\} (27)$$

Finally, the aggregated score $({AS}_{i})$ for each alternative is computed using:

$$\left\{ \begin{aligned} {AS}_{i}=\frac{1}{2}\left( {NSP}_{i}+{NSN}_{i} \right) \\ 0\leq{AS}_{i}\leq1 \forall i\in\left\{ 1.2\ldots.m \right\} \end{aligned} \right\} (28)$$

The alternatives are ranked based on their $({AS}_{i})$ values, where a higher score indicates a more favorable alternative. The EDAS method provides a robust prioritization mechanism by accounting for both positive and negative deviations from the average, ensuring a comprehensive evaluation of other options.

In this study, an attempt has been made to determine the criteria weights using the CRITIC approach and then to evaluate and rank countries' performance within each cluster using the three methods: MACONT, COCOSO, and EDAS. Using the same techniques, appropriate distinctions between clusters have been established, with each cluster represented by the mean across all criteria in that cluster.

**Appendix B. Country Rankings by Cluster and Pandemic Phase**

This appendix reports the detailed country-level ranking results within each cluster for each pandemic phase. Tables B.1-B.3 present the rankings obtained from individual MCDM methods (MACONT, COCOSO, and EDAS), along with their corresponding scores and aggregated ranks. These detailed results are provided for completeness and transparency, while the main text focuses on comparative patterns and key insights.

**Table B.1 .** Countries ranking in each cluster according to the decision-making methods in the Pre-Vaccination Phase

| Cluster 1 |  |  |  |  | Cluster 2 |  |  |  |  | Cluster 3 |  |  |  |  |
| --- | --- | --- | --- | --- | --- | --- | --- | --- | --- | --- | --- | --- | --- | --- |
| Country | MACONT | COCOSO | EDAS | Final Rank | Country | MACONT | COCOSO | EDAS | Final Rank | Country | MACONT | COCOSO | EDAS | Final Rank |
| United States | 0.329078 | 0.054846 | 0.767668 | 1 | Namibia | 0.344776 | 0.057463 | 0.727574 | 1 | Brazil | 0.329489 | 0.054915 | 0.735164 | 1 |
| Singapore | 0.36367 | 0.060612 | 0.555977 | 2 | Ghana | 0.325313 | 0.054219 | 0.576353 | 2 | Mexico | 0.349795 | 0.058299 | 0.665915 | 2 |
| Germany | 0.318951 | 0.053159 | 0.587974 | 3 | Rwanda | 0.324402 | 0.054067 | 0.573554 | 3 | Kazakhstan | 0.375576 | 0.062596 | 0.609811 | 3 |
| Sweden | 0.360738 | 0.060123 | 0.527346 | 4 | Eswatini | 0.299194 | 0.049866 | 0.596195 | 4 | Turkey | 0.363169 | 0.060528 | 0.622562 | 4 |
| Malta | 0.282494 | 0.047082 | 0.617949 | 5 | Pakistan | 0.282802 | 0.047134 | 0.596588 | 5 | Argentina | 0.345841 | 0.05764 | 0.628461 | 5 |
| Australia | 0.330227 | 0.055038 | 0.561567 | 6 | Sao Tome and Principe | 0.307762 | 0.051294 | 0.550261 | 6 | Cuba | 0.346957 | 0.057826 | 0.564915 | 6 |
| Canada | 0.294637 | 0.049106 | 0.594287 | 7 | Nigeria | 0.273862 | 0.045644 | 0.587148 | 7 | Costa Rica | 0.360718 | 0.06012 | 0.546476 | 7 |
| Luxembourg | 0.309807 | 0.051634 | 0.559867 | 8 | Sudan | 0.278372 | 0.046395 | 0.579213 | 8 | Maldives | 0.312396 | 0.052066 | 0.59911 | 8 |
| Denmark | 0.348657 | 0.058109 | 0.494978 | 9 | Senegal | 0.300831 | 0.050138 | 0.546339 | 9 | Bahamas | 0.313766 | 0.052294 | 0.561564 | 9 |
| Oman | 0.297036 | 0.049506 | 0.550828 | 10 | Lesotho | 0.265405 | 0.044234 | 0.5818 | 10 | Romania | 0.315105 | 0.052518 | 0.555328 | 10 |
| Japan | 0.314095 | 0.052349 | 0.530792 | 11 | Kenya | 0.263511 | 0.043918 | 0.575583 | 11 | Iran | 0.320872 | 0.053479 | 0.548531 | 11 |
| Bahrain | 0.301832 | 0.050305 | 0.543095 | 12 | Cambodia | 0.298008 | 0.049668 | 0.514265 | 12 | Azerbaijan | 0.325168 | 0.054195 | 0.532226 | 12 |
| Spain | 0.306501 | 0.051084 | 0.530689 | 13 | Nepal | 0.259782 | 0.043297 | 0.541531 | 13 | Armenia | 0.327888 | 0.054648 | 0.529038 | 13 |
| Norway | 0.343054 | 0.057176 | 0.48405 | 14 | Angola | 0.260581 | 0.04343 | 0.538681 | 14 | Mauritius | 0.339474 | 0.056579 | 0.515219 | 14 |
| Netherlands | 0.308934 | 0.051489 | 0.513372 | 15 | Mauritania | 0.266661 | 0.044443 | 0.526027 | 15 | Thailand | 0.322035 | 0.053672 | 0.52519 | 15 |
| New Zealand | 0.321509 | 0.053585 | 0.496941 | 16 | Zambia | 0.264928 | 0.044155 | 0.527677 | 16 | Barbados | 0.320879 | 0.05348 | 0.525567 | 16 |
| Belgium | 0.291686 | 0.048614 | 0.52814 | 17 | Zimbabwe | 0.26281 | 0.043802 | 0.521435 | 17 | Georgia | 0.306143 | 0.051024 | 0.538128 | 17 |
| Saudi Arabia | 0.286327 | 0.047721 | 0.528059 | 18 | Cameroon | 0.265365 | 0.044227 | 0.512545 | 18 | India | 0.245263 | 0.040877 | 0.607675 | 18 |
| Switzerland | 0.301009 | 0.050168 | 0.510311 | 19 | Congo | 0.259435 | 0.043239 | 0.514201 | 19 | Jordan | 0.308847 | 0.051474 | 0.533395 | 19 |
| Malaysia | 0.30203 | 0.050338 | 0.508093 | 20 | Guinea-Bissau | 0.210319 | 0.035053 | 0.555001 | 20 | Ecuador | 0.285607 | 0.047601 | 0.559085 | 20 |
| Qatar | 0.295489 | 0.049248 | 0.514014 | 21 | Gambia | 0.254296 | 0.042383 | 0.496352 | 21 | Algeria | 0.301373 | 0.050229 | 0.539885 | 21 |
| Finland | 0.306441 | 0.051073 | 0.501169 | 22 | Ethiopia | 0.224388 | 0.037398 | 0.517933 | 22 | Panama | 0.309552 | 0.051592 | 0.529624 | 22 |
| United Kingdom | 0.28564 | 0.047607 | 0.514692 | 23 | Uganda | 0.224564 | 0.037427 | 0.503568 | 23 | Seychelles | 0.328493 | 0.054749 | 0.486014 | 23 |
| Iceland | 0.302073 | 0.050345 | 0.4873 | 24 | Solomon Islands | 0.235862 | 0.03931 | 0.485898 | 24 | North Macedonia | 0.312622 | 0.052104 | 0.501115 | 24 |
| Portugal | 0.287168 | 0.047861 | 0.5031 | 25 | Mali | 0.234626 | 0.039104 | 0.485919 | 25 | Trinidad and Tobago | 0.302963 | 0.050494 | 0.501049 | 25 |
| China | 0.244514 | 0.040752 | 0.534421 | 26 | Djibouti | 0.233975 | 0.038996 | 0.471031 | 26 | Montenegro | 0.310982 | 0.05183 | 0.491602 | 26 |
| Kuwait | 0.278813 | 0.046469 | 0.489711 | 27 | Liberia | 0.228488 | 0.038081 | 0.476794 | 27 | Egypt | 0.289442 | 0.04824 | 0.512301 | 27 |
| France | 0.253609 | 0.042268 | 0.512123 | 28 | Mozambique | 0.218571 | 0.036429 | 0.478232 | 28 | South Africa | 0.245867 | 0.040978 | 0.53981 | 28 |
| Italy | 0.268025 | 0.044671 | 0.486846 | 29 | Yemen | 0.203907 | 0.033985 | 0.479248 | 29 | Morocco | 0.275113 | 0.045852 | 0.505608 | 29 |
| Estonia | 0.268986 | 0.044831 | 0.471321 | 30 | Sierra Leone | 0.219211 | 0.036535 | 0.459792 | 30 | Albania | 0.293186 | 0.048864 | 0.483193 | 30 |
| Hungary | 0.25101 | 0.041835 | 0.490763 | 31 | Guinea | 0.216842 | 0.03614 | 0.457613 | 31 | Bhutan | 0.260618 | 0.043436 | 0.516223 | 31 |
| Lithuania | 0.269182 | 0.044864 | 0.46786 | 32 | Togo | 0.204962 | 0.03416 | 0.445834 | 32 | Antigua and Barbuda | 0.285147 | 0.047524 | 0.47069 | 32 |
| Uruguay | 0.278605 | 0.046434 | 0.44507 | 33 | Afghanistan | 0.176903 | 0.029484 | 0.474866 | 33 | Serbia | 0.276577 | 0.046096 | 0.479595 | 33 |
| Slovenia | 0.259181 | 0.043197 | 0.45571 | 34 | Malawi | 0.205075 | 0.034179 | 0.42877 | 34 | Tunisia | 0.284791 | 0.047465 | 0.469238 | 34 |
| Cyprus | 0.260124 | 0.043354 | 0.454035 | 35 | Benin | 0.191883 | 0.03198 | 0.427289 | 35 | Suriname | 0.26657 | 0.044428 | 0.490025 | 35 |
| Chile | 0.249488 | 0.041581 | 0.446925 | 36 | Haiti | 0.156627 | 0.026105 | 0.457709 | 36 | Ukraine | 0.253259 | 0.04221 | 0.501549 | 36 |
| Slovakia | 0.239041 | 0.03984 | 0.4583 | 37 | Madagascar | 0.178582 | 0.029764 | 0.423988 | 37 | Botswana | 0.256959 | 0.042827 | 0.496481 | 37 |
| Latvia | 0.244547 | 0.040758 | 0.450063 | 38 | Burkina Faso | 0.182169 | 0.030362 | 0.416291 | 38 | Fiji | 0.275585 | 0.045931 | 0.472311 | 38 |
| Poland | 0.242016 | 0.040336 | 0.445001 | 39 | Niger | 0.173464 | 0.028911 | 0.411316 | 39 | Indonesia | 0.245935 | 0.040989 | 0.504686 | 39 |
| Russia | 0.279466 | 0.046578 | 0.39607 | 40 | Comoros | 0.172481 | 0.028747 | 0.401752 | 40 | Jamaica | 0.272118 | 0.045353 | 0.471237 | 40 |
| Austria | 0.219 | 0.0365 | 0.459732 | 41 | Chad | 0.140879 | 0.02348 | 0.431181 | 41 | Lebanon | 0.255634 | 0.042606 | 0.486973 | 41 |
| Greece | 0.241484 | 0.040247 | 0.426675 | 42 | South Sudan | 0.164113 | 0.027352 | 0.298123 | 42 | Peru | 0.245306 | 0.040884 | 0.485623 | 42 |
| Ireland | 0.215591 | 0.035932 | 0.426859 | 43 | Central African Republic | 0.097467 | 0.016245 | 0.328533 | 43 | El Salvador | 0.263008 | 0.043835 | 0.460636 | 43 |
| Bulgaria | 0.191371 | 0.031895 | 0.425908 | 44 |  |  |  |  |  | Mongolia | 0.263725 | 0.043954 | 0.459585 | 44 |
| Belarus | 0.223943 | 0.037324 | 0.37834 | 45 |  |  |  |  |  | Kyrgyzstan | 0.243157 | 0.040526 | 0.479588 | 45 |
| Croatia | 0.219386 | 0.036564 | 0.36601 | 46 |  |  |  |  |  | Saint Lucia | 0.257695 | 0.042949 | 0.459305 | 46 |
|  |  |  |  |  |  |  |  |  |  | Paraguay | 0.249111 | 0.041518 | 0.464223 | 47 |
|  |  |  |  |  |  |  |  |  |  | Philippines | 0.239848 | 0.039975 | 0.471623 | 48 |
|  |  |  |  |  |  |  |  |  |  | Dominican Republic | 0.245286 | 0.040881 | 0.452675 | 49 |
|  |  |  |  |  |  |  |  |  |  | Sri Lanka | 0.257892 | 0.042982 | 0.436458 | 50 |
|  |  |  |  |  |  |  |  |  |  | Guyana | 0.234032 | 0.039005 | 0.454574 | 51 |
|  |  |  |  |  |  |  |  |  |  | Libya | 0.227002 | 0.037834 | 0.448751 | 52 |
|  |  |  |  |  |  |  |  |  |  | Uzbekistan | 0.238728 | 0.039788 | 0.433426 | 53 |
|  |  |  |  |  |  |  |  |  |  | Gabon | 0.213186 | 0.035531 | 0.462265 | 54 |
|  |  |  |  |  |  |  |  |  |  | Iraq | 0.202491 | 0.033749 | 0.455518 | 55 |
|  |  |  |  |  |  |  |  |  |  | Belize | 0.212201 | 0.035367 | 0.433021 | 56 |
|  |  |  |  |  |  |  |  |  |  | Tajikistan | 0.215294 | 0.035882 | 0.429156 | 57 |
|  |  |  |  |  |  |  |  |  |  | Myanmar | 0.182451 | 0.030408 | 0.464395 | 58 |
|  |  |  |  |  |  |  |  |  |  | Bosnia and Herzegovina | 0.220371 | 0.036729 | 0.414353 | 59 |
|  |  |  |  |  |  |  |  |  |  | Guatemala | 0.200106 | 0.033351 | 0.420794 | 60 |
|  |  |  |  |  |  |  |  |  |  | Bangladesh | 0.195547 | 0.032591 | 0.42484 | 61 |
|  |  |  |  |  |  |  |  |  |  | Honduras | 0.193616 | 0.032269 | 0.411326 | 62 |
|  |  |  |  |  |  |  |  |  |  | Colombia | 0.324169 | 0.054028 | 0.243196 | 63 |
|  |  |  |  |  |  |  |  |  |  | Nicaragua | 0.19942 | 0.033237 | 0.382102 | 64 |

**Table B.2.** Countries ranking in each cluster according to the decision-making methods in the Vaccination Phase

| Cluster 1 |  |  |  |  | Cluster 2 |  |  |  |  | Cluster 3 |  |  |  |  |
| --- | --- | --- | --- | --- | --- | --- | --- | --- | --- | --- | --- | --- | --- | --- |
| Country | MACONT | COCOSO | EDAS | Final Rank | Country | MACONT | COCOSO | EDAS | Final Rank | Country | MACONT | COCOSO | EDAS | Final Rank |
| United States | 0.318803 | 0.053134 | 0.701031 | 1 | Rwanda | 0.321209 | 0.053535 | 0.650817 | 1 | Antigua and Barbuda | 0.29254 | 0.048757 | 0.820354 | 1 |
| Singapore | 0.375893 | 0.062649 | 0.575611 | 2 | Namibia | 0.277994 | 0.046332 | 0.6826 | 2 | Seychelles | 0.328688 | 0.054781 | 0.753233 | 2 |
| Malta | 0.30286 | 0.050477 | 0.63584 | 3 | Gabon | 0.292749 | 0.048792 | 0.613335 | 3 | Saudi Arabia | 0.378419 | 0.06307 | 0.652523 | 3 |
| Australia | 0.334127 | 0.055688 | 0.583411 | 4 | Pakistan | 0.270775 | 0.045129 | 0.622034 | 4 | Brazil | 0.32618 | 0.054363 | 0.704341 | 4 |
| Germany | 0.320468 | 0.053411 | 0.583897 | 5 | Comoros | 0.197915 | 0.032986 | 0.651288 | 5 | Oman | 0.370978 | 0.06183 | 0.614689 | 5 |
| Canada | 0.301719 | 0.050287 | 0.574779 | 6 | Eswatini | 0.251076 | 0.041846 | 0.583012 | 6 | Mexico | 0.329736 | 0.054956 | 0.637151 | 6 |
| Denmark | 0.351091 | 0.058515 | 0.516697 | 7 | Ghana | 0.278258 | 0.046376 | 0.550323 | 7 | Argentina | 0.345035 | 0.057506 | 0.617294 | 7 |
| Japan | 0.318991 | 0.053165 | 0.540824 | 8 | Sao Tome and Principe | 0.261988 | 0.043665 | 0.546762 | 8 | Kuwait | 0.36555 | 0.060925 | 0.592059 | 8 |
| Sweden | 0.347612 | 0.057935 | 0.499876 | 9 | Solomon Islands | 0.220602 | 0.036767 | 0.589724 | 9 | Kazakhstan | 0.338481 | 0.056413 | 0.574177 | 9 |
| New Zealand | 0.321466 | 0.053578 | 0.525085 | 10 | Sudan | 0.244391 | 0.040732 | 0.547022 | 10 | Turkey | 0.333159 | 0.055527 | 0.579743 | 10 |
| Luxembourg | 0.298405 | 0.049734 | 0.546303 | 11 | Lesotho | 0.223464 | 0.037244 | 0.552191 | 11 | Costa Rica | 0.354599 | 0.0591 | 0.548825 | 11 |
| Norway | 0.34401 | 0.057335 | 0.492657 | 12 | Kenya | 0.217327 | 0.036221 | 0.542772 | 12 | Mauritius | 0.330829 | 0.055138 | 0.576138 | 12 |
| Spain | 0.313652 | 0.052275 | 0.520168 | 13 | Zimbabwe | 0.230333 | 0.038389 | 0.525078 | 13 | Barbados | 0.295332 | 0.049222 | 0.594555 | 13 |
| Bahrain | 0.310379 | 0.05173 | 0.522421 | 14 | Nigeria | 0.217462 | 0.036244 | 0.534693 | 14 | Maldives | 0.299319 | 0.049887 | 0.582936 | 14 |
| Finland | 0.314062 | 0.052344 | 0.511063 | 15 | Djibouti | 0.21564 | 0.03594 | 0.521139 | 15 | Thailand | 0.315239 | 0.05254 | 0.543704 | 15 |
| Iceland | 0.311649 | 0.051942 | 0.511984 | 16 | Zambia | 0.218494 | 0.036416 | 0.513874 | 16 | Iran | 0.300693 | 0.050115 | 0.551823 | 16 |
| Netherlands | 0.31123 | 0.051872 | 0.511258 | 17 | Mauritania | 0.222901 | 0.03715 | 0.50836 | 17 | Ecuador | 0.288543 | 0.04809 | 0.561601 | 17 |
| Portugal | 0.302739 | 0.050456 | 0.51656 | 18 | Senegal | 0.238755 | 0.039792 | 0.489421 | 18 | Bhutan | 0.280958 | 0.046826 | 0.550627 | 18 |
| Malaysia | 0.30773 | 0.051288 | 0.506445 | 19 | Congo | 0.223714 | 0.037286 | 0.506558 | 19 | Bulgaria | 0.29268 | 0.04878 | 0.521695 | 19 |
| Belgium | 0.300021 | 0.050003 | 0.515358 | 20 | Angola | 0.212968 | 0.035495 | 0.511011 | 20 | Panama | 0.297055 | 0.049509 | 0.513005 | 20 |
| Qatar | 0.309309 | 0.051552 | 0.499537 | 21 | Liberia | 0.223758 | 0.037293 | 0.496092 | 21 | Montenegro | 0.285943 | 0.047657 | 0.516543 | 21 |
| Cyprus | 0.281564 | 0.046927 | 0.527499 | 22 | Ethiopia | 0.202017 | 0.03367 | 0.506126 | 22 | Azerbaijan | 0.299253 | 0.049875 | 0.500801 | 22 |
| China | 0.267091 | 0.044515 | 0.530663 | 23 | Gambia | 0.21418 | 0.035697 | 0.487126 | 23 | India | 0.235543 | 0.039257 | 0.559761 | 23 |
| Switzerland | 0.290647 | 0.048441 | 0.494609 | 24 | Mali | 0.217032 | 0.036172 | 0.481749 | 24 | Peru | 0.265058 | 0.044176 | 0.520296 | 24 |
| Italy | 0.286289 | 0.047715 | 0.486175 | 25 | Mozambique | 0.196938 | 0.032823 | 0.494135 | 25 | Bahamas | 0.266047 | 0.044341 | 0.517654 | 25 |
| France | 0.264806 | 0.044134 | 0.506706 | 26 | Uganda | 0.195924 | 0.032654 | 0.490516 | 26 | Romania | 0.273236 | 0.045539 | 0.506145 | 26 |
| Uruguay | 0.289101 | 0.048183 | 0.476538 | 27 | Guinea-Bissau | 0.172514 | 0.028752 | 0.516392 | 27 | Georgia | 0.273852 | 0.045642 | 0.496151 | 27 |
| United Kingdom | 0.282399 | 0.047066 | 0.481172 | 28 | Cameroon | 0.211365 | 0.035228 | 0.447018 | 28 | Trinidad and Tobago | 0.264841 | 0.04414 | 0.500847 | 28 |
| Chile | 0.286948 | 0.047825 | 0.465893 | 29 | Yemen | 0.179131 | 0.029855 | 0.467034 | 29 | Mongolia | 0.261025 | 0.043504 | 0.498025 | 29 |
| Austria | 0.234333 | 0.039056 | 0.501766 | 30 | Sierra Leone | 0.189184 | 0.031531 | 0.449066 | 30 | Armenia | 0.286443 | 0.04774 | 0.465964 | 30 |
| Cuba | 0.26655 | 0.044425 | 0.462468 | 31 | Guinea | 0.19131 | 0.031885 | 0.440656 | 31 | Jordan | 0.27241 | 0.045402 | 0.478881 | 31 |
| Hungary | 0.246502 | 0.041084 | 0.481278 | 32 | Togo | 0.175699 | 0.029283 | 0.446135 | 32 | Fiji | 0.264594 | 0.044099 | 0.483154 | 32 |
| Estonia | 0.25639 | 0.042732 | 0.468149 | 33 | Benin | 0.180898 | 0.03015 | 0.430922 | 33 | North Macedonia | 0.276265 | 0.046044 | 0.464264 | 33 |
| Lithuania | 0.25779 | 0.042965 | 0.458488 | 34 | Afghanistan | 0.154726 | 0.025788 | 0.460662 | 34 | Morocco | 0.262417 | 0.043736 | 0.480285 | 34 |
| Greece | 0.249001 | 0.0415 | 0.45031 | 35 | Malawi | 0.173007 | 0.028834 | 0.437021 | 35 | Serbia | 0.260385 | 0.043397 | 0.46434 | 35 |
| Latvia | 0.239191 | 0.039865 | 0.461293 | 36 | Niger | 0.170504 | 0.028417 | 0.420516 | 36 | Botswana | 0.235563 | 0.03926 | 0.491198 | 36 |
| Slovenia | 0.249914 | 0.041652 | 0.444503 | 37 | Haiti | 0.14018 | 0.023363 | 0.453175 | 37 | Indonesia | 0.236452 | 0.039409 | 0.49003 | 37 |
| Ireland | 0.236549 | 0.039425 | 0.44125 | 38 | Burkina Faso | 0.170365 | 0.028394 | 0.395104 | 38 | Algeria | 0.249471 | 0.041578 | 0.474527 | 38 |
| Slovakia | 0.225474 | 0.037579 | 0.451529 | 39 | Madagascar | 0.151015 | 0.025169 | 0.407971 | 39 | Albania | 0.262984 | 0.043831 | 0.455675 | 39 |
| Poland | 0.231517 | 0.038586 | 0.417109 | 40 | Chad | 0.125464 | 0.020911 | 0.409793 | 40 | Tunisia | 0.257911 | 0.042985 | 0.45955 | 40 |
| Russia | 0.261675 | 0.043613 | 0.377587 | 41 | South Sudan | 0.133952 | 0.022325 | 0.296787 | 41 | Egypt | 0.254605 | 0.042434 | 0.459515 | 41 |
| Belarus | 0.224373 | 0.037396 | 0.366892 | 42 | Central African Republic | 0.089934 | 0.014989 | 0.323992 | 42 | Sri Lanka | 0.258936 | 0.043156 | 0.453969 | 42 |
| Croatia | 0.207926 | 0.034654 | 0.357316 | 43 |  |  |  |  |  | Suriname | 0.233667 | 0.038944 | 0.47748 | 43 |
|  |  |  |  |  |  |  |  |  |  | El Salvador | 0.260746 | 0.043458 | 0.437058 | 44 |
|  |  |  |  |  |  |  |  |  |  | South Africa | 0.214459 | 0.035743 | 0.490234 | 45 |
|  |  |  |  |  |  |  |  |  |  | Ukraine | 0.228518 | 0.038086 | 0.467609 | 46 |
|  |  |  |  |  |  |  |  |  |  | Philippines | 0.234221 | 0.039037 | 0.457213 | 47 |
|  |  |  |  |  |  |  |  |  |  | Guyana | 0.216893 | 0.036149 | 0.475994 | 48 |
|  |  |  |  |  |  |  |  |  |  | Lebanon | 0.229821 | 0.038303 | 0.4547 | 49 |
|  |  |  |  |  |  |  |  |  |  | Paraguay | 0.235152 | 0.039192 | 0.445894 | 50 |
|  |  |  |  |  |  |  |  |  |  | Uzbekistan | 0.237132 | 0.039522 | 0.44126 | 51 |
|  |  |  |  |  |  |  |  |  |  | Jamaica | 0.233389 | 0.038898 | 0.443917 | 52 |
|  |  |  |  |  |  |  |  |  |  | Saint Lucia | 0.222113 | 0.037019 | 0.443614 | 53 |
|  |  |  |  |  |  |  |  |  |  | Dominican Republic | 0.231036 | 0.038506 | 0.43283 | 54 |
|  |  |  |  |  |  |  |  |  |  | Cambodia | 0.200815 | 0.033469 | 0.466312 | 55 |
|  |  |  |  |  |  |  |  |  |  | Tajikistan | 0.221991 | 0.036998 | 0.42794 | 56 |
|  |  |  |  |  |  |  |  |  |  | Kyrgyzstan | 0.213078 | 0.035513 | 0.437691 | 57 |
|  |  |  |  |  |  |  |  |  |  | Bangladesh | 0.206725 | 0.034454 | 0.426121 | 58 |
|  |  |  |  |  |  |  |  |  |  | Nicaragua | 0.215674 | 0.035946 | 0.415677 | 59 |
|  |  |  |  |  |  |  |  |  |  | Myanmar | 0.182732 | 0.030455 | 0.452139 | 60 |
|  |  |  |  |  |  |  |  |  |  | Belize | 0.201963 | 0.03366 | 0.416944 | 61 |
|  |  |  |  |  |  |  |  |  |  | Honduras | 0.19591 | 0.032652 | 0.41366 | 62 |
|  |  |  |  |  |  |  |  |  |  | Guatemala | 0.185529 | 0.030921 | 0.407995 | 63 |
|  |  |  |  |  |  |  |  |  |  | Libya | 0.18653 | 0.031088 | 0.406037 | 64 |
|  |  |  |  |  |  |  |  |  |  | Colombia | 0.309308 | 0.051551 | 0.245044 | 65 |
|  |  |  |  |  |  |  |  |  |  | Bosnia and Herzegovina | 0.190626 | 0.031771 | 0.38343 | 66 |
|  |  |  |  |  |  |  |  |  |  | Nepal | 0.16054 | 0.026757 | 0.403705 | 67 |
|  |  |  |  |  |  |  |  |  |  | Iraq | 0.162351 | 0.027059 | 0.401453 | 68 |

**Table B.3.** Countries ranking in each cluster according to the decision-making methods in the Post-Vaccination Phase

| Cluster 1 |  |  |  |  | Cluster 2 |  |  |  |  | Cluster 3 |  |  |  |  |
| --- | --- | --- | --- | --- | --- | --- | --- | --- | --- | --- | --- | --- | --- | --- |
| Country | MACONT | COCOSO | EDAS | Final Rank | Country | MACONT | COCOSO | EDAS | Final Rank | Country | MACONT | COCOSO | EDAS | Final Rank |
| China | 0.284223 | 0.047371 | 0.940888 | 1 | Pakistan | 0.300936 | 0.050156 | 0.876062 | 1 | India | 0.254302 | 0.042384 | 1.004347 | 1 |
| United States | 0.333376 | 0.055563 | 0.788573 | 2 | Namibia | 0.29575 | 0.049292 | 0.661706 | 2 | Brazil | 0.291496 | 0.048583 | 0.817726 | 2 |
| Singapore | 0.355037 | 0.059173 | 0.556885 | 3 | Nigeria | 0.251796 | 0.041966 | 0.676569 | 3 | Saudi Arabia | 0.347679 | 0.057947 | 0.651414 | 3 |
| Australia | 0.328494 | 0.054749 | 0.572545 | 4 | Gabon | 0.305495 | 0.050916 | 0.606338 | 4 | Mexico | 0.304162 | 0.050694 | 0.680385 | 4 |
| Germany | 0.311903 | 0.051984 | 0.5893 | 5 | Ghana | 0.293384 | 0.048897 | 0.567423 | 5 | Bahrain | 0.344694 | 0.057449 | 0.610935 | 5 |
| Japan | 0.304844 | 0.050807 | 0.584423 | 6 | Rwanda | 0.295134 | 0.049189 | 0.563784 | 6 | Malaysia | 0.342488 | 0.057081 | 0.611972 | 6 |
| Malta | 0.276812 | 0.046135 | 0.613227 | 7 | Cambodia | 0.275283 | 0.04588 | 0.580177 | 7 | Oman | 0.350434 | 0.058406 | 0.595032 | 7 |
| Sweden | 0.357573 | 0.059595 | 0.515758 | 8 | Sudan | 0.252874 | 0.042146 | 0.564188 | 8 | Argentina | 0.297868 | 0.049645 | 0.602359 | 8 |
| Canada | 0.288164 | 0.048027 | 0.579679 | 9 | Nepal | 0.248845 | 0.041474 | 0.559078 | 9 | Turkey | 0.307773 | 0.051295 | 0.590777 | 9 |
| Luxembourg | 0.304725 | 0.050788 | 0.541802 | 10 | Eswatini | 0.258592 | 0.043099 | 0.545501 | 10 | Kuwait | 0.327047 | 0.054508 | 0.557096 | 10 |
| Denmark | 0.342763 | 0.057127 | 0.488139 | 11 | Kenya | 0.235922 | 0.03932 | 0.565882 | 11 | Kazakhstan | 0.319153 | 0.053192 | 0.553232 | 11 |
| New Zealand | 0.319921 | 0.05332 | 0.498946 | 12 | Senegal | 0.263822 | 0.04397 | 0.520951 | 12 | Cuba | 0.30386 | 0.050643 | 0.543548 | 12 |
| Norway | 0.337221 | 0.056204 | 0.478348 | 13 | Ethiopia | 0.21842 | 0.036403 | 0.570207 | 13 | Maldives | 0.269112 | 0.044852 | 0.564904 | 13 |
| Finland | 0.298298 | 0.049716 | 0.507271 | 14 | Sao Tome and Principe | 0.260414 | 0.043402 | 0.516631 | 14 | Iran | 0.271645 | 0.045274 | 0.556944 | 14 |
| Spain | 0.29687 | 0.049478 | 0.501588 | 15 | Lesotho | 0.230249 | 0.038375 | 0.536677 | 15 | Mauritius | 0.28565 | 0.047608 | 0.539449 | 15 |
| Netherlands | 0.301611 | 0.050268 | 0.48872 | 16 | Zambia | 0.23079 | 0.038465 | 0.519368 | 16 | Costa Rica | 0.307099 | 0.051183 | 0.513178 | 16 |
| Belgium | 0.284948 | 0.047491 | 0.491894 | 17 | Zimbabwe | 0.233568 | 0.038928 | 0.503991 | 17 | Thailand | 0.277671 | 0.046278 | 0.537931 | 17 |
| Qatar | 0.290378 | 0.048396 | 0.485441 | 18 | Angola | 0.224444 | 0.037407 | 0.509531 | 18 | Barbados | 0.271397 | 0.045233 | 0.527416 | 18 |
| United Kingdom | 0.282916 | 0.047153 | 0.492981 | 19 | Mauritania | 0.230613 | 0.038435 | 0.498296 | 19 | Indonesia | 0.223358 | 0.037226 | 0.582396 | 19 |
| Iceland | 0.29776 | 0.049627 | 0.472946 | 20 | Congo | 0.225428 | 0.037571 | 0.501079 | 20 | Belarus | 0.293662 | 0.048944 | 0.490305 | 20 |
| Switzerland | 0.293213 | 0.048869 | 0.474503 | 21 | Cameroon | 0.233828 | 0.038971 | 0.480294 | 21 | Seychelles | 0.277635 | 0.046272 | 0.504434 | 21 |
| Portugal | 0.275856 | 0.045976 | 0.472099 | 22 | Guinea-Bissau | 0.184176 | 0.030696 | 0.536819 | 22 | Azerbaijan | 0.27589 | 0.045982 | 0.502081 | 22 |
| France | 0.250796 | 0.041799 | 0.498505 | 23 | Mali | 0.221457 | 0.036909 | 0.488731 | 23 | Bahamas | 0.258641 | 0.043107 | 0.521788 | 23 |
| Estonia | 0.264591 | 0.044098 | 0.474311 | 24 | Gambia | 0.22869 | 0.038115 | 0.464669 | 24 | Ecuador | 0.25112 | 0.041853 | 0.528955 | 24 |
| Uruguay | 0.284235 | 0.047373 | 0.450939 | 25 | Uganda | 0.207962 | 0.03466 | 0.482544 | 25 | Armenia | 0.286399 | 0.047733 | 0.485697 | 25 |
| Italy | 0.259437 | 0.04324 | 0.471802 | 26 | Mozambique | 0.20413 | 0.034022 | 0.481602 | 26 | Romania | 0.258656 | 0.043109 | 0.513335 | 26 |
| Lithuania | 0.267802 | 0.044634 | 0.453567 | 27 | Liberia | 0.211529 | 0.035255 | 0.469998 | 27 | Algeria | 0.255622 | 0.042604 | 0.509072 | 27 |
| Hungary | 0.242723 | 0.040454 | 0.468591 | 28 | Solomon Islands | 0.213215 | 0.035536 | 0.465351 | 28 | Georgia | 0.267484 | 0.044581 | 0.481796 | 28 |
| Russia | 0.286573 | 0.047762 | 0.415967 | 29 | Djibouti | 0.205416 | 0.034236 | 0.449509 | 29 | Jordan | 0.268811 | 0.044802 | 0.478523 | 29 |
| Cyprus | 0.257092 | 0.042849 | 0.440023 | 30 | Guinea | 0.203501 | 0.033917 | 0.442426 | 30 | Panama | 0.263752 | 0.043959 | 0.482463 | 30 |
| Slovenia | 0.251745 | 0.041958 | 0.44221 | 31 | Yemen | 0.182733 | 0.030455 | 0.466266 | 31 | Montenegro | 0.263516 | 0.043919 | 0.476858 | 31 |
| Latvia | 0.243876 | 0.040646 | 0.449258 | 32 | Afghanistan | 0.165855 | 0.027643 | 0.485129 | 32 | Trinidad and Tobago | 0.250014 | 0.041669 | 0.490387 | 32 |
| Chile | 0.250503 | 0.04175 | 0.426077 | 33 | Sierra Leone | 0.192573 | 0.032096 | 0.452718 | 33 | Egypt | 0.250538 | 0.041756 | 0.481215 | 33 |
| Greece | 0.230776 | 0.038463 | 0.426663 | 34 | Togo | 0.187842 | 0.031307 | 0.443228 | 34 | North Macedonia | 0.267727 | 0.044621 | 0.460262 | 34 |
| Slovakia | 0.234175 | 0.039029 | 0.422627 | 35 | Malawi | 0.188222 | 0.03137 | 0.422886 | 35 | Morocco | 0.241091 | 0.040182 | 0.489993 | 35 |
| Poland | 0.2365 | 0.039417 | 0.419745 | 36 | Niger | 0.167548 | 0.027925 | 0.426353 | 36 | Bhutan | 0.227677 | 0.037946 | 0.505501 | 36 |
| Austria | 0.210709 | 0.035118 | 0.444751 | 37 | Benin | 0.175118 | 0.029186 | 0.41194 | 37 | South Africa | 0.210975 | 0.035163 | 0.520936 | 37 |
| Ireland | 0.216008 | 0.036001 | 0.41239 | 38 | Madagascar | 0.167721 | 0.027953 | 0.413681 | 38 | Albania | 0.251267 | 0.041878 | 0.455793 | 38 |
| Bulgaria | 0.185092 | 0.030849 | 0.396729 | 39 | Haiti | 0.138892 | 0.023149 | 0.442692 | 39 | Tunisia | 0.247532 | 0.041255 | 0.459685 | 39 |
| Croatia | 0.210811 | 0.035135 | 0.34989 | 40 | Burkina Faso | 0.169257 | 0.028209 | 0.404651 | 40 | Fiji | 0.248534 | 0.041422 | 0.455038 | 40 |
|  |  |  |  |  | Comoros | 0.148045 | 0.024674 | 0.399854 | 41 | Mongolia | 0.229946 | 0.038324 | 0.471619 | 41 |
|  |  |  |  |  | Chad | 0.120834 | 0.020139 | 0.421597 | 42 | Serbia | 0.232443 | 0.038741 | 0.467062 | 42 |
|  |  |  |  |  | South Sudan | 0.141288 | 0.023548 | 0.272699 | 43 | Ukraine | 0.222304 | 0.037051 | 0.478524 | 43 |
|  |  |  |  |  | Central African Republic | 0.085872 | 0.014312 | 0.330922 | 44 | Philippines | 0.212544 | 0.035424 | 0.488737 | 44 |
|  |  |  |  |  |  |  |  |  |  | Antigua and Barbuda | 0.238643 | 0.039774 | 0.456623 | 45 |
|  |  |  |  |  |  |  |  |  |  | Peru | 0.213877 | 0.035646 | 0.485135 | 46 |
|  |  |  |  |  |  |  |  |  |  | Suriname | 0.230523 | 0.03842 | 0.456043 | 47 |
|  |  |  |  |  |  |  |  |  |  | Kyrgyzstan | 0.221863 | 0.036977 | 0.462323 | 48 |
|  |  |  |  |  |  |  |  |  |  | Botswana | 0.218196 | 0.036366 | 0.461303 | 49 |
|  |  |  |  |  |  |  |  |  |  | Jamaica | 0.238971 | 0.039828 | 0.436415 | 50 |
|  |  |  |  |  |  |  |  |  |  | Saint Lucia | 0.223954 | 0.037326 | 0.447925 | 51 |
|  |  |  |  |  |  |  |  |  |  | Lebanon | 0.213886 | 0.035648 | 0.457193 | 52 |
|  |  |  |  |  |  |  |  |  |  | Bangladesh | 0.184123 | 0.030687 | 0.487087 | 53 |
|  |  |  |  |  |  |  |  |  |  | Sri Lanka | 0.22586 | 0.037643 | 0.436424 | 54 |
|  |  |  |  |  |  |  |  |  |  | Uzbekistan | 0.214935 | 0.035822 | 0.446467 | 55 |
|  |  |  |  |  |  |  |  |  |  | Paraguay | 0.224686 | 0.037448 | 0.431812 | 56 |
|  |  |  |  |  |  |  |  |  |  | El Salvador | 0.23509 | 0.039182 | 0.416364 | 57 |
|  |  |  |  |  |  |  |  |  |  | Guyana | 0.205978 | 0.03433 | 0.44454 | 58 |
|  |  |  |  |  |  |  |  |  |  | Myanmar | 0.162824 | 0.027137 | 0.476043 | 59 |
|  |  |  |  |  |  |  |  |  |  | Dominican Republic | 0.207587 | 0.034598 | 0.409823 | 60 |
|  |  |  |  |  |  |  |  |  |  | Tajikistan | 0.196408 | 0.032735 | 0.414013 | 61 |
|  |  |  |  |  |  |  |  |  |  | Iraq | 0.179695 | 0.029949 | 0.42797 | 62 |
|  |  |  |  |  |  |  |  |  |  | Libya | 0.198888 | 0.033148 | 0.404571 | 63 |
|  |  |  |  |  |  |  |  |  |  | Guatemala | 0.183187 | 0.030531 | 0.422211 | 64 |
|  |  |  |  |  |  |  |  |  |  | Bosnia and Herzegovina | 0.192211 | 0.032035 | 0.406086 | 65 |
|  |  |  |  |  |  |  |  |  |  | Belize | 0.183763 | 0.030627 | 0.402279 | 66 |
|  |  |  |  |  |  |  |  |  |  | Nicaragua | 0.18412 | 0.030687 | 0.384062 | 67 |
|  |  |  |  |  |  |  |  |  |  | Honduras | 0.177367 | 0.029561 | 0.391636 | 68 |
|  |  |  |  |  |  |  |  |  |  | Colombia | 0.285435 | 0.047573 | 0.194551 | 69 |

**Appendix C. Criteria Weights Across Pandemic Phases**

This appendix reports the complete set of criteria weights derived using the CRITIC method for each pandemic phase. Tables C.1-C.3 present the phase-specific weights corresponding to the pre-vaccination, vaccination, and post-vaccination periods. These detailed results support the robustness of the weighting process, while key patterns and influential criteria are discussed in the main paper.

**Table** **C.1.** Criteria importance in each cluster in the Pre-Vaccination Phase

| Criterion | Cluster 1 | Cluster 2 | Cluster 3 |
| --- | --- | --- | --- |
| Estimated number of people living with HIV - Adult (>15) rate | 1 | 29 | 39 |
| Adult literacy rate, population 15+ years | 2 | 19 | 12 |
| Operational readiness index | 3 | 2 | 3 |
| Net ODA received (% of GNI) | 4 | 31 | 7 |
| The proportion of the target population with access to 3 doses of diphtheria-tetanus-pertussis (DTP3) (%) | 5 | 23 | 22 |
| Average of 13 International Health Regulations core capacity scores, SPAR version | 6 | 27 | 19 |
| Population living in urban areas (%) | 7 | 5 | 6 |
| IHR capacity score: Points of entry | 8 | 3 | 1 |
| Total Cases | 9 | 32 | 8 |
| Attitudes towards violence | 10 | 36 | 2 |
| Public trust in politicians | 11 | 41 | 5 |
| Gender Inequality Index | 12 | 15 | 16 |
| International tourism, number of arrivals | 13 | 46 | 20 |
| Volume of remittances (in USD) as a proportion of total GDP (%) | 14 | 18 | 23 |
| The proportion of the target population with access to measles-containing-vaccine second-dose (MCV2) (%) | 15 | 8 | 11 |
| Total Deaths | 16 | 47 | 45 |
| People practicing open defecation (% of the population) | 17 | 4 | 42 |
| Diabetes Prevalence | 18 | 30 | 35 |
| People using at least basic sanitation services (% of population) | 19 | 6 | 15 |
| Income Gini coefficient | 20 | 7 | 26 |
| Corruption Perception Index | 21 | 28 | 14 |
| Individuals using the Internet (% of the population) | 22 | 12 | 10 |
| 1+ underlying conditions plus 0 conditions (65+ yrs) | 23 | 39 | 9 |
| Life Expectancy | 24 | 20 | 29 |
| Government Effectiveness | 25 | 42 | 40 |
| Prevalence of Undernourishment | 26 | 11 | 28 |
| Mobile cellular subscriptions | 27 | 13 | 17 |
| Children under 5 (% of population) | 28 | 38 | 24 |
| Stringency Index | 29 | 22 | 37 |
| Hospital Beds Per Thousand | 30 | 1 | 18 |
| Human Development Index | 31 | 33 | 32 |
| Physicians Density | 32 | 10 | 43 |
| Maternal Mortality Ratio | 33 | 17 | 25 |
| Land area (sq. km) | 34 | 35 | 44 |
| The proportion of the target population with access to pneumococcal conjugate 3rd dose (PCV3) (%) | 35 | 26 | 38 |
| People using at least basic drinking water services (% of population) | 36 | 24 | 4 |
| Reproduction Rate | 37 | 21 | 27 |
| Refugees and asylum-seekers by country of asylum | 38 | 9 | 36 |
| Urban population growth (annual %) | 39 | 37 | 41 |
| GDP Per Capita | 40 | 40 | 13 |
| Current health expenditure per capita | 41 | 45 | 33 |
| Air transport, passengers carried | 42 | 34 | 31 |
| Access to Cities | 43 | 43 | 21 |
| Internally displaced persons (IDPs) | 44 | 25 | 46 |
| Population Density | 45 | 16 | 30 |
| Road length | 46 | 14 | 34 |
| Returned Refugees | 47 | 48 | 47 |
| Number of people requiring interventions against neglected tropical diseases | 48 | 44 | 48 |

**Table C.2.** Criteria importance in each cluster in the Vaccination Phase

| Criterion | Cluster 1 | Cluster 2 | Cluster 3 |
| --- | --- | --- | --- |
| Estimated number of people living with HIV - Adult (>15) rate | 1 | 32 | 45 |
| Adult literacy rate, population 15+ years | 2 | 21 | 16 |
| Operational readiness index | 3 | 1 | 4 |
| Net ODA received (% of GNI) | 4 | 30 | 3 |
| Average of 13 International Health Regulations core capacity scores, SPAR version | 5 | 31 | 25 |
| The proportion of the target population with access to 3 doses of diphtheria-tetanus-pertussis (DTP3) (%) | 6 | 22 | 33 |
| IHR capacity score: Points of entry | 7 | 7 | 1 |
| International tourism, number of arrivals | 8 | 54 | 24 |
| PERSONS_BOOSTER_ADD_DOSE_PER100 | 9 | 3 | 10 |
| Public trust in politicians | 10 | 46 | 13 |
| Population living in urban areas (%) | 11 | 20 | 11 |
| Gender Inequality Index | 12 | 17 | 22 |
| The proportion of the target population with access to measles-containing-vaccine second-dose (MCV2) (%) | 13 | 8 | 9 |
| Attitudes towards violence | 14 | 37 | 2 |
| People practicing open defecation (% of the population) | 15 | 6 | 44 |
| Volume of remittances (in USD) as a proportion of total GDP (%) | 16 | 34 | 20 |
| Total Deaths | 17 | 51 | 21 |
| Total Cases | 18 | 39 | 18 |
| Income Gini coefficient | 19 | 12 | 28 |
| Diabetes Prevalence | 20 | 28 | 34 |
| Corruption Perception Index | 21 | 27 | 17 |
| Government Effectiveness | 22 | 43 | 46 |
| Human Development Index | 23 | 44 | 30 |
| 1+ underlying conditions plus 0 conditions (65+ yrs) | 24 | 42 | 14 |
| Children under 5 (% of population) | 25 | 47 | 29 |
| Land area (sq. km) | 26 | 36 | 51 |
| Hospital Beds Per Thousand | 27 | 49 | 26 |
| Life Expectancy | 28 | 29 | 40 |
| Positive Rate | 29 | 4 | 19 |
| Refugees and asylum-seekers by country of asylum | 30 | 10 | 43 |
| People using at least basic sanitation services (% of population) | 31 | 13 | 23 |
| The proportion of the target population with access to pneumococcal conjugate 3rd dose (PCV3) (%) | 32 | 23 | 47 |
| Maternal Mortality Ratio | 33 | 18 | 31 |
| Prevalence of Undernourishment | 34 | 11 | 32 |
| Total Tests | 35 | 35 | 48 |
| Individuals using the Internet (% of the population) | 36 | 38 | 8 |
| Mobile cellular subscriptions | 37 | 16 | 15 |
| Total Vaccinations | 38 | 45 | 7 |
| Total Boosters | 39 | 41 | 53 |
| Current health expenditure per capita | 40 | 52 | 37 |
| People using at least basic drinking water services (% of population) | 41 | 26 | 12 |
| GDP Per Capita | 42 | 53 | 49 |
| Stringency Index | 43 | 9 | 42 |
| Air transport, passengers carried | 44 | 33 | 35 |
| Urban population growth (annual %) | 45 | 40 | 36 |
| Physicians Density | 46 | 14 | 41 |
| Access to Cities | 47 | 48 | 27 |
| People Fully Vaccinated | 48 | 25 | 6 |
| Internally displaced persons (IDPs) | 49 | 24 | 52 |
| Road length | 50 | 15 | 39 |
| Reproduction Rate | 51 | 5 | 50 |
| FIRST VACCINE DATE | 52 | 2 | 5 |
| Population Density | 53 | 19 | 38 |
| Returned Refugees | 54 | 55 | 54 |
| Number of people requiring interventions against neglected tropical diseases | 55 | 50 | 55 |

**Table C.3.** Criteria importance in each cluster in the Post-Vaccination Phase

| Criterion | Cluster 1 | Cluster 2 | Cluster 3 |
| --- | --- | --- | --- |
| Estimated number of people living with HIV - Adult (>15) rate | 1 | 24 | 40 |
| Adult literacy rate, population 15+ years | 2 | 17 | 12 |
| The proportion of the target population with access to 3 doses of diphtheria-tetanus-pertussis (DTP3) (%) | 3 | 22 | 24 |
| Operational readiness index | 4 | 2 | 17 |
| Average of 13 International Health Regulations core capacity scores, SPAR version | 5 | 26 | 11 |
| IHR capacity score: Points of entry | 6 | 3 | 2 |
| Public trust in politicians | 7 | 40 | 7 |
| Population living in urban areas (%) | 8 | 12 | 9 |
| Total Cases | 9 | 46 | 8 |
| International tourism, number of arrivals | 10 | 48 | 15 |
| Children under 5 (% of population) | 11 | 38 | 22 |
| Net ODA received (% of GNI) | 12 | 29 | 4 |
| Gender Inequality Index | 13 | 14 | 14 |
| Volume of remittances (in USD) as a proportion of total GDP (%) | 14 | 18 | 18 |
| Total Deaths | 15 | 33 | 30 |
| Mobile cellular subscriptions | 16 | 8 | 16 |
| The proportion of the target population with access to measles-containing-vaccine second-dose (MCV2) (%) | 17 | 7 | 10 |
| Income Gini coefficient | 18 | 6 | 20 |
| People practicing open defecation (% of the population) | 19 | 4 | 45 |
| People using at least basic sanitation services (% of population) | 20 | 5 | 26 |
| Government Effectiveness | 21 | 39 | 41 |
| Individuals using the Internet (% of the population) | 22 | 32 | 25 |
| Corruption Perception Index | 23 | 20 | 13 |
| Urban population growth (annual %) | 24 | 36 | 23 |
| Physicians Density | 25 | 10 | 43 |
| Attitudes towards violence | 26 | 31 | 1 |
| Life Expectancy | 27 | 21 | 32 |
| The proportion of the target population with access to pneumococcal conjugate 3rd dose (PCV3) (%) | 28 | 27 | 39 |
| Refugees and asylum-seekers by country of asylum | 29 | 11 | 38 |
| Land area (sq. km) | 30 | 34 | 44 |
| Hospital Beds Per Thousand | 31 | 42 | 34 |
| 1+ underlying conditions plus 0 conditions (65+ yrs) | 32 | 35 | 6 |
| Reproduction Rate | 33 | 1 | 3 |
| GDP Per Capita | 34 | 45 | 42 |
| Human Development Index | 35 | 37 | 31 |
| Diabetes Prevalence | 36 | 28 | 21 |
| Current health expenditure per capita | 37 | 43 | 28 |
| Access to Cities | 38 | 41 | 19 |
| Internally displaced persons (IDPs) | 39 | 25 | 47 |
| Maternal Mortality Ratio | 40 | 13 | 33 |
| Air transport, passengers carried | 41 | 30 | 36 |
| Population Density | 42 | 15 | 35 |
| Road length | 43 | 16 | 37 |
| Returned Refugees | 44 | 51 | 48 |
| Prevalence of Undernourishment | 45 | 9 | 27 |
| People using at least basic drinking water services (% of population) | 46 | 23 | 5 |
| Stringency Index | 47 | 19 | 29 |
| Total Boosters | 48 | 50 | 46 |
| Number of people requiring interventions against neglected tropical diseases | 49 | 47 | 50 |
| Total Vaccinations | 50 | 49 | 49 |
| People Fully Vaccinated | 51 | 44 | 51 |
